# Epidemiological trends of lung cancer attributed to residential radon exposure at global, regional, and national level: a trend analysis study from 1990 to 2021

**DOI:** 10.3389/fpubh.2025.1593415

**Published:** 2025-05-02

**Authors:** Qiang Xiong, Zhen Zhang, Jianliang Peng, Jing Liang, Dexing Lian, Xipeng Zhao, Lulu Wang, Tiangxiang Lu, Yuwen Li

**Affiliations:** ^1^National Center for Occupational Safety and Health, National Health Commission of the People’s Republic of China, Beijing, China; ^2^Key Laboratory for Engineering Control of Dust Hazard, National Health Commission of the People’s Republic of China, Beijing, China

**Keywords:** lung cancer, residential radon, global burden, epidemiology, trend analysis

## Abstract

**Background:**

Lung cancer (LC) remains a leading cause of cancer-related mortality globally, with radon identified as the second major risk factor. This study aimed to analyze the global, regional, and national burden of LC attributed to residential radon exposure from 1990 to 2021.

**Methods:**

The Global Burden of Disease (GBD) 2021 database were employed to estimate the disease trends of LC attributed to residential radon exposure across sex, age groups, and socioeconomic development levels via the socio-demographic index (SDI). Trends of the age-standardized rates (ASRs) were evaluated using estimated annual percentage change (EAPC). The relationship of the socio-demographic index (SDI) with ASRs was assessed via Spearman correlation and LOESS regression.

**Results:**

In 2021, residential radon caused 82,160 global LC deaths (an increase of 66.87% since 1990), while the ASRs declined globally (ASMR EAPC: −0.26, 95%C: −0.51 to −0.01; ASDR EAPC: −0.65, 95%CI: −0.85 to −0.44). The disease burden of residential radon-induced LC was higher in middle and high latitude nations. With the increase of SDI, ASRs showed a downward trend in most regions, while an upward trend at national level. Across age and sex, the older adult males exhibited higher burden.

**Conclusion:**

While global ASRs declined, rising absolute burdens underscore radon’s persistent threat, particularly in rapidly urbanizing and high-latitude regions. Targeted radon mitigation, enhanced early detection, and gender-specific interventions are critical.

## Introduction

Lung cancer (LC) remains a leading cause cancer-related deaths globally, with an estimated 2.5 million new cases and 1.80 million deaths according to GLOBOCAN 2022 (1), underscoring its critical public health implications. The World Health Organization (WHO) identifies residential radon exposure as the second most significant risk factor for LC, contributing to 10–20% of cases and 3–20% of fatalities, following tobacco use (2).

Radon is a radioactive gas that is both colorless and odorless, often referred to as the “silent killer.” It is estimated to be responsible for up to 50% of human radiation exposure (3). Originating from geological substrates such as granite, brick, sand, cement, and gypsum, particularly from natural stones that contain radioactive elements, residential radon accumulates in enclosed indoor environments, where prolonged exposure occurs (4, 5). Research indicates that the average concentration of residential radon globally is approximately 50 Bq/m^3^, with levels in high radon regions reaching up to 200 Bq/m^3^ (6, 7). The International Commission of Radiological Protection (ICRP) has stated that that a radon concentration of 300 Bq/m^3^ in indoors equates to an annual radiation dose of 14 mSv, exceeding that of a full chest computed tomography (CT) scan (8).

Epidemiological studies demonstrated a dose–response relationship between radon exposure and LC risk. Meta-analyses indicated an 16% increase in LC incidence per 100 Bq/m^3^ rise in radon levels (9). Similarly, European research has indicated a 0.08 increase in excess relative risk of LC corresponding to each 100 Bq/m^3^ elevation in residential radon levels (10), while Chinese studies report relative risks of 1.01 per 10 Bq/m^3^ increments in indoor radon levels (11). These findings underscore the imperative to mitigate residential radon exposure as a critical public health intervention. Consequently, this research employed the most recent Global Burden of Disease (GBD) data to systematically analyze the global burden of LC attributed to residential radon exposure from 1990 to 2021, comparing the distribution and variations across demographic and geographic strata.

## Materials and methods

### Data source

This analysis utilized a subset of the GBD 2021 database to investigate the disease burden of LC attributable to residential radon exposure between 1990 and 2021. The GBD 2021 database, accessible through the website of the Institute for Health Metrics and Evaluation (IHME),^1^ provides estimates of disease burden for 204 countries, encompassing 371 diseases and injuries as well as 88 risk factors from 1990 to 2021 (12–14). The estimation of relative risk (RR) for risk factors was based on data derived from 3,359 primary randomized controlled trials, cohort studies, case–control studies, and pertinent meta-analyses from diverse countries. These data were compiled via a systematic literature review to ensure comprehensive and accurate information. Additionally, to estimate exposure levels for risk factors, 51,337 unique data sources were integrated, including household and health surveys, population censuses, ground monitoring data, and administrative records (13).

### Estimation of residential radon exposure

The estimation process for residential radon exposure in this study adhered to a systematic methodology (13). Firstly, comprehensive epidemiological data covering 204 countries and regions were compiled. Secondly, a systematic literature review method was employed to estimate the RR between residential radon exposure and LC. The burden of proof risk function (BPRF) method was utilized to address heterogeneity among various studies, thereby facilitating a more conservative interpretation of the association between residential radon and LC. Thirdly, the theoretical minimum risk exposure level (TMREL) for residential radon was calculated, serving as an ideal exposure standard for assessing health risk reduction. Based on the obtained RR and TMREL, the population attribution fraction (PAF) was computed to quantify the proportion of health risk alterations attributed to reductions in exposure to TMREL. Finally, the attributable burden of residential radon was estimated based on the disease burden of PAF and associated health outcomes, measured in disability-adjusted life years (DALYs), and presented stratified by age, gender, geographic location, and time to achieve clarity in results.

### Estimated annual percentage change and percentage change

A generalized linear regression model was employed to compute the estimated annual percentage change (EAPC) of the age-standardized rates (ASRs), which was subsequently used to determine the ASRs trend of residential radon-induced LC from 1990 to 2021. This model calculated EAPC and 95% CI by establishing an equation between the natural logarithm of ASRs and the year. The calculation formula was: $\mathrm{EAPC}=\left(\exp\left(\beta \right)-1\right)\times 100\text{,}$ where *β* represented the regression coefficient of the year independent variable in the time regression model.

Percentage changes were utilized to reflect the trend of LC deaths and DALYs number attributable to residential radon from 1990 to 2021, calculated using the formula: $\mathrm{percentage}\;\mathrm{change}=\left(\mathrm{numbe}r_{2021}-\mathrm{numbe}r_{1990}\right)/\mathrm{numbe}r_{1990}\text{.}$

### Association of SDI and ASRs

In the GBD 2021 framework, multiple indicators were employed to calculate the socio-demographic index (SDI), including the total fertility rate under the age of 25 (TFU 25), mean education for those ages 15 and older (EDU 15+), and lag distributed income (LDI) per capita. Firstly, data pertaining to GDP, educational attainment rates, and total fertility rates of various nations were collected, with missing values appropriately addressed and outliers processed. Principal component analysis (PCA) was subsequently performed on the standardized data to ascertain the relative positioning of each country regarding social determinants. Ultimately, SDI values were categorized into five levels (high SDI, high-middle SDI, middle SDI, low-middle SDI, and low SDI), reflecting disparities in socio-economic development and health outcomes across different regions. The numerical range of SDI spanned from 0 to 1, with 0 indicating the lowest level of development and 1 signifying the highest level. SDI data for various countries and regions could be obtained from the IHME website.^2^ The correlation between SDI and ASRs was evaluated through Spearman correlation analysis, while the expected relationships were explored using local weighted regression (LOESS) models.

All data cleaning, statistical analysis, and visualization in this study were conducted using R software (version 4.3.3).

## Results

### Global mortality and DALYs

In 2021, residential radon exposure accounted for 82,160 (95% UI: −41,645 to 210,377) global LC deaths, representing a 66.87% increase from 1990 levels (Table 1). However, the age-standardized mortality rate (ASMR) exhibited a downward trend, decreasing from 1.26 (95% UI: −0.61 to 3.22) per 100,000 population in 1990 to 0.96 (95% UI: −0.48 to 2.45) per 100,000 in 2021, with an EAPC of −0.89 (95% CI: −0.94 to −0.84). DALYs reached 189,805 (95% UI: −968,774 to 4,852,214) in 2021, marking a 46.18% increase compared to 1990 (Supplementary Table S1). Concurrently, the age-standardized DALY rate (ASDR) declined from 31.51 (95% UI: −15.22 to 80.26) per 100,000 in 1990 to 21.73 (95% UI: −11.08 to 55.55) per 100,000 in 2021, corresponding to an EAPC of −0.65 (95% CI: −0.85 to −0.44).

**Table 1:** The mortality number and rate of lung cancer attributed to residential radon exposure in 1990 and 2021 across 21 regions and global, and the trends from 1990 to 2021.

Region	All ages mortality number			Age-standard mortality rate per 100,000 population		
	1990 (95% UI)	2021 (95% UI)	Percentage change, %	1990 (95% UI)	2021 (95% UI)	EAPC (95% CI)
Global	49,237 (−23,789, 125,873)	82,160 (−41,645, 210,377)	66.87	1.26 (−0.61, 3.22)	0.96 (−0.48, 2.45)	−0.89 (−0.94, −0.84)
Andean Latin America	111 (−52, 389)	260 (−123, 881)	133.70	0.56 (−0.26,1.97)	0.45 (−0.21, 1.52)	−0.84 (−1.01, −0.67)
Australasia	117 (−53, 389)	178 (−85, 575)	52.29	0.49 (−0.22, 1.63)	0.32 (−0.15, 1.03)	−1.36 (−1.40, −1.31)
Caribbean	113 (−40, 377)	211 (−78, 659)	87.08	0.44 (−0.15, 1.48)	0.39 (−0.15, 1.22)	−0.17 (−0.25, −0.09)
Central Asia	1,008 (−438, 2,936)	811 (−353, 2,264)	−19.56	2.07 (−0.90, 6.05)	0.99 (−0.44, 2.76)	−2.13 (−2.24, −2.02)
Central Europe	3,457 (−1,638, 8,874)	4,486 (−2,208, 11,489)	29.76	2.27 (−1.07, 5.82)	2.01 (−0.99, 5.14)	−0.37 (−0.46, −0.29)
Central Latin America	624 (−280, 1,625)	1,250 (−523, 3,378)	100.28	0.79 (−0.36, 2.04)	0.51 (−0.21, 1.37)	−1.62 (−1.69, −1.55)
Central Sub-Saharan Africa	84 (−36, 317)	190 (−101, 754)	127.82	0.38 (−0.17, 1.47)	0.36 (−0.19, 1.40)	−0.21 (−0.43, 0.01)
East Asia	10,516 (−5,138, 28,403)	30,411 (−15,416, 83,590)	189.18	1.26 (−0.61, 3.41)	1.40 (−0.71, 3.85)	0.40 (0.23, 0.58)
Eastern Europe	6,850 (−3,298, 17,746)	5,108 (−2,524, 13,134)	−25.43	2.38 (−1.14, 6.16)	1.43 (−0.71, 3.67)	−1.83 (−1.95, −1.71)
Eastern Sub-Saharan Africa	216 (−90, 578)	410 (−174, 1,091)	89.73	0.29 (−0.12, 0.78)	0.26 (−0.11, 0.69)	−0.53 (−0.64, −0.42)
High-income Asia Pacific	1,142 (−564, 3,067)	2,773 (−1,457, 7,695)	142.88	0.57 (−0.28, 1.53)	0.54 (−0.28, 1.51)	−0.34 (−0.46, −0.21)
High-income North America	7,664 (−3,574, 20,030)	8,593 (−4,091, 21,881)	12.12	2.19 (−1.02, 5.72)	1.26 (−0.60, 3.20)	−1.91 (−2.10, −1.72)
North Africa and Middle East	1,216 (−510, 3,572)	2,788 (−1,255, 7,669)	129.21	0.74 (−0.31, 2.17)	0.65 (−0.29, 1.79)	−0.3 (−0.44, −0.16)
Oceania	16 (−6, 54)	41 (−18, 152)	165.07	0.57 (−0.23, 1.93)	0.59 (−0.26, 2.17)	0.20 (0.14, 0.27)
South Asia	1,542 (−733, 3,968)	4,356 (−2,062, 11,277)	182.52	0.27 (−0.13, 0.70)	0.29 (−0.14, 0.76)	0.17 (0.07, 0.27)
Southeast Asia	987 (−471, 2,842)	2,671 (−1,199, 7,541)	172.24	0.40 (−0.19, 1.14)	0.42 (−0.19, 1.19)	−0.03 (−0.12, 0.06)
Southern Latin America	379 (−152, 1,304)	493 (−201, 1,663)	30.14	0.82 (−0.33, 2.81)	0.56 (−0.23, 1.89)	−1.05 (−1.14, −0.95)
Southern Sub-Saharan Africa	213 (−106, 600)	505 (−232, 1,474)	137.20	0.79 (−0.39, 2.21)	0.89 (−0.41, 2.60)	0.31 (0.02, 0.60)
Tropical Latin America	643 (−297, 1,760)	1,621 (−753, 4,554)	152.01	0.72 (−0.33, 1.96)	0.63 (−0.29, −1.78)	−0.39 (−0.46, −0.31)
Western Europe	12,211 (−5,957, 32,193)	14,661 (−6,859, 38,255)	20.06	2.12 (−1.03, 5.58)	1.56 (−0.73, 4.07)	−0.85 (−0.91, −0.78)
Western Sub-Saharan Africa	134 (−60, 347)	343 (−153, 885)	156.17	0.16 (−0.07, 0.41)	0.19 (−0.08, 0.49)	0.77 (0.70, 0.84)

UI, uncertainty intervals; EAPC, estimated annual percentage change.

### Regional mortality and DALYs

East Asia (30,411, 95% UI: −15,416 to 83,590), Western Europe (14,661, 95% UI: −6,859 to 38,255), and high-income North America (8,593, 95% UI: −4,091 to 21,881) exhibited the highest mortality in 2021 (Table 1). With the exceptions of Eastern Europe (−25.43%) and Central Asia (−19.56%), most regions exhibited increased mortality of residential radon-induced LC from 1990 to 2021. The most substantial percentage increases were observed in East Asia (189.18%), South Asia (182.52%), and Southeast Asia (172.24%). In terms of ASMR, Central Europe (2.01/100,000, 95% UI: −0.99/100,000 to 5.14/100,000), Western Europe (1.56/100,000, 95% UI: −0.73/100,000 to 4.07/100,000), and Eastern Europe (1.43/100,000, 95% UI: −0.71/100,000 to 3.67/100,000) demonstrated the highest values in 2021. Notably, with the exceptions of Western Sub-Saharan Africa, East Asia, Southern Sub-Saharan Africa, Oceania, and South Asia, all other regions experienced declining ASMR trends compared to 1990. The most pronounced reductions in ASMR were identified in Central Asia (EAPC: −2.13, 95% CI: −2.24 to −2.02), high-income North America (EAPC: −1.91, 95% CI: −2.10 to −1.72), and Eastern Europe (EAPC: −1.83, 95% CI: −1.95 to −1.71). DALYs number and ASDR associated with residential radon-related LC in 2021 exhibited comparable epidemiological patterns to mortality data (Supplementary Table S1).

### National mortality and DALYs

**Figure 1:**
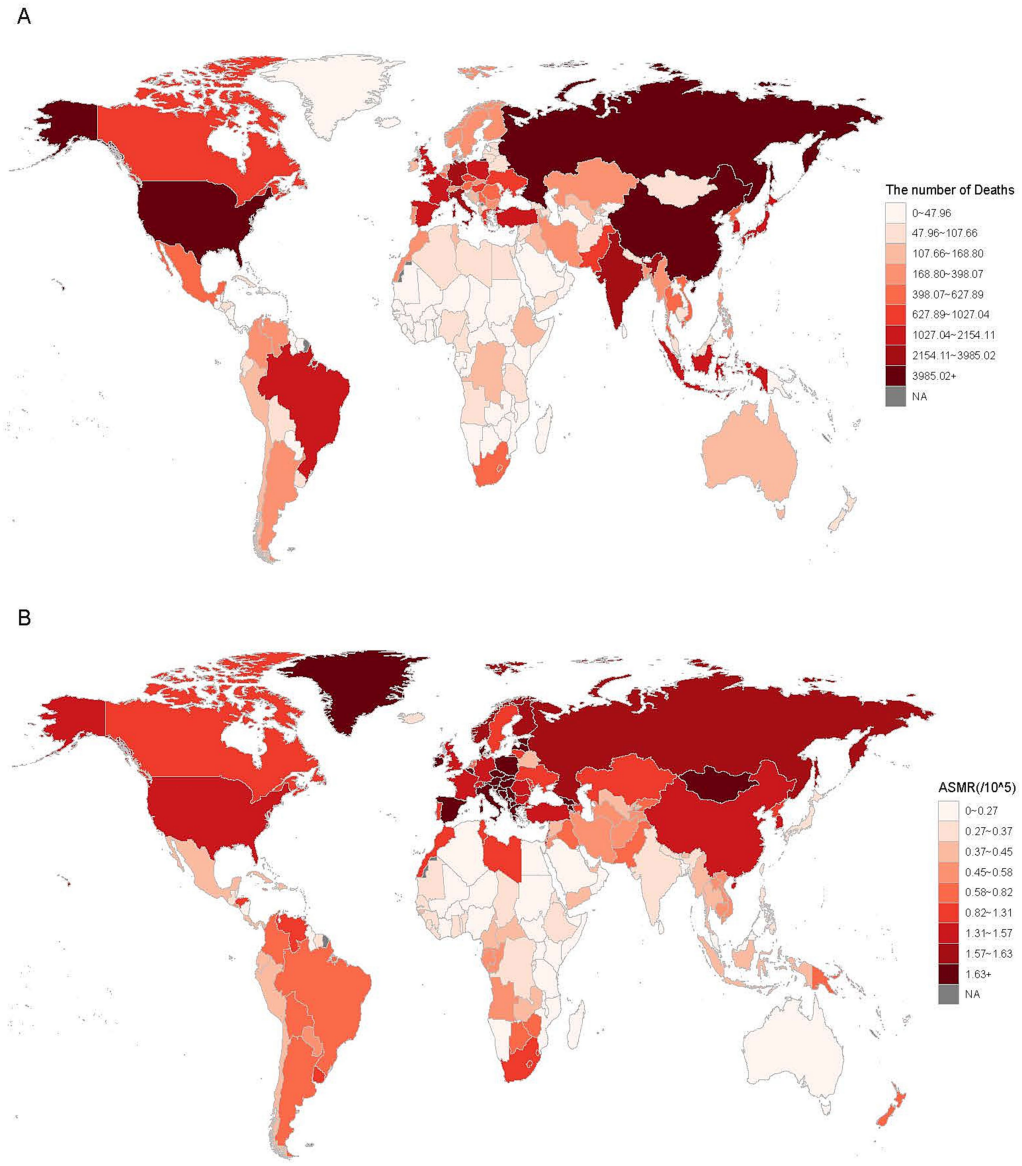
The mortality **(A)** and ASMR **(B)** of LC attributed to residential radon exposure at global in 204 countries in 2021. ASMR, age-standardized mortality rate.

In 2021, the three countries with the highest deaths number of LC attributable to residential radon exposure were China (29,890, 95% UI: −15,235 to 82,113), the United States (7,961, 95% UI: −3,751 to 20,761), and Russia (3,985, 95% UI: −1,972 to 10,415, Figure 1). The most substantial reductions in mortality of radon-related LC were observed in Brazil (−89.42%), Ukraine (−51.34%), and Kazakhstan (−48.34%). Conversely, Djibouti (515.95%), Egypt (439.45%), and the United Arab Emirates (385.23%) exhibited the most pronounced mortality increases (Table 2). However, it is noteworthy that these countries had completely low baseline mortality estimates in 1990. Regarding ASMR, Greenland (4.91/100,000, 95% UI: −2.05/100,000 to 18.28/100,000), Hungary (3.39/100,000, 95% UI: −1.38/100,000 to 9.83/100,000), and Armenia (3.01/100,000, 95% UI: −1.51/100,000 to 9.62/100,000) demonstrated the highest values in 2021 (Figure 1). Approximately 60% of countries exhibited declining ASMR trends from 1990 to 2021, with the most rapid decreases observed in Kazakhstan (EAPC: −3.24, 95% CI: −3.41 to −3.06), Ukraine (EAPC: −2.80, 95% CI: −3.00 to −2.61), and Uzbekistan (EAPC: −2.72, 95% CI: −3.03 to −2.40). In contrast, the most marked ASMR increases were identified in Egypt (EAPC: 4.11, 95% CI: 3.56–4.68), Lesotho (EAPC: 2.85, 95% CI: 2.50–3.20), and Kenya (EAPC: 1.79, 95% CI: 1.61–1.97, Table 2). At the national level, DALYs number and ASDR associated with residential radon-related LC in 2021 demonstrated similar trends observed in mortality data (Supplementary Figure S1 and Supplementary Table S2).

**Table 2:** The mortality number and rate of lung cancer attributed to residential radon exposure in 1990 and 2021 across 204 countries, and the trends from 1990 to 2021.

Location	All ages mortality number			Age-standard mortality rate per 100,000 population		
	1990 (95% UI)	2021 (95% UI)	Percentage change, %	1990 (95% UI)	2021 (95% UI)	EAPC (95% CI)
Afghanistan	31 (−11, 134)	49 (−19, 199)	58.12	0.45 (−0.17, 1.95)	0.51 (−0.21, 2.02)	0.58 (0.46, 0.71)
Albania	66 (−35, 182)	120 (−66, 347)	82.50	3.23 (−1.74, 8.98)	2.69 (−1.48, 7.75)	−0.39 (−0.62, −0.16)
Algeria	33 (−13, 130)	80 (−30, 308)	141.97	0.3 (−0.12, 1.2)	0.24 (−0.09, 0.93)	−0.43 (−0.66, −0.20)
American Samoa	0 (0, 1)	0 (0, 1)	100.30	0.85 (−0.28, 3.59)	0.77 (−0.27, 3.18)	−0.20 (−0.26, −0.14)
Andorra	1 (−1, 6)	2 (−1, 8)	41.98	2.56 (−0.97, 10.02)	1.36 (−0.58, 5.34)	−1.74 (−2.03, −1.46)
Angola	19 (−8, 79)	53 (−21, 248)	183.28	0.49 (−0.2, 2.09)	0.47 (−0.18, 2.19)	−0.13 (−0.27, 0)
Antigua and Barbuda	0 (0, 1)	0 (0, 1)	66.24	0.28 (−0.09, 1.17)	0.24 (−0.08, 0.97)	−0.28 (−0.55, −0.02)
Argentina	276 (−111, 1,094)	328 (−132, 1,247)	18.93	0.85 (−0.34, 3.37)	0.58 (−0.23, 2.21)	−1.04 (−1.17, −0.91)
Armenia	114 (−57, 375)	133 (−67, 425)	16.68	3.94 (−1.96, 13)	3.01 (−1.51, 9.62)	−0.62 (−0.89, −0.36)
Australia	75 (−31, 269)	118 (−51, 423)	58.31	0.38 (−0.16, 1.36)	0.25 (−0.11, 0.91)	−1.3 (−1.34, −1.25)
Austria	341 (−150, 941)	406 (−178, 1,130)	19.16	2.9 (−1.29, 8.03)	2.26 (−0.99, 6.27)	−0.51 (−0.61, −0.41)
Azerbaijan	71 (−28, 294)	94 (−39, 369)	33.11	1.33 (−0.53, 5.58)	0.87 (−0.36, 3.44)	−0.92 (−1.21, −0.64)
Bahamas	1 (0, 3)	2 (−1, 7)	117.94	0.45 (−0.16, 2.14)	0.38 (−0.12, 1.7)	−0.28 (−0.41, −0.15)
Bahrain	2 (−1, 7)	4 (−2, 20)	161.13	1.12 (−0.47, 4.88)	0.61 (−0.24, 2.78)	−2.47 (−2.78, −2.16)
Bangladesh	127 (−52, 498)	267 (−122, 969)	109.73	0.27 (−0.11, 1.07)	0.2 (−0.09, 0.7)	−1.13 (−1.27, −0.98)
Barbados	1 (0, 4)	1 (−1, 6)	60.24	0.29 (−0.1, 1.39)	0.26 (−0.1, 1.21)	−0.13 (−0.26, 0)
Belarus	74 (−31, 241)	61 (−26, 213)	−16.85	0.56 (−0.23, 1.82)	0.38 (−0.16, 1.29)	−2.02 (−2.29, −1.76)
Belgium	459 (−178, 1,413)	423 (−162, 1,351)	−7.88	2.98 (−1.15, 9.2)	1.81 (−0.69, 5.73)	−1.39 (−1.55, −1.23)
Belize	0 (0, 1)	1 (0, 4)	244.63	0.3 (−0.14, 1.36)	0.32 (−0.16, 1.52)	0.29 (−0.22, 0.80)
Benin	5 (−2, 23)	13 (−4, 53)	144.40	0.28 (−0.09, 1.21)	0.27 (−0.09, 1.1)	0.17 (0.02, 0.31)
Bermuda	1 (0, 4)	1 (0, 4)	26.60	1.33 (−0.49, 5.75)	0.74 (−0.27, 3.04)	−1.65 (−1.82, −1.49)
Bhutan	1 (0, 3)	2 (−1, 8)	171.32	0.34 (−0.16, 1.41)	0.38 (−0.2, 1.41)	0.49 (0.38, 0.61)
Bolivia (Plurinational State of)	24 (−7, 95)	58 (−19, 249)	145.51	0.77 (−0.24, 3.1)	0.67 (−0.22, 2.85)	−0.39 (−0.48, −0.30)
Bosnia and Herzegovina	78 (−36, 323)	116 (−56, 484)	48.23	1.82 (−0.84, 7.49)	1.83 (−0.88, 7.64)	0.14 (0.02, 0.27)
Botswana	4 (−1, 16)	9 (−3, 39)	139.10	0.69 (−0.25, 2.93)	0.64 (−0.24, 2.69)	−0.47 (−0.80, −0.15)
Brazil	637 (−295, 1,743)	67 (−30, 228)	−89.42	0.73 (−0.34, 1.99)	0.64 (−0.3, 1.79)	−0.42 (−0.49, −0.35)
Brunei Darussalam	0 (0, 2)	1 (0, 4)	117.08	0.42 (−0.14, 1.95)	0.28 (−0.09, 1.24)	−0.86 (−1.07, −0.64)
Bulgaria	153 (−71, 486)	193 (−87, 588)	26.13	1.23 (−0.57, 3.89)	1.41 (−0.63, 4.29)	0.82 (0.68, 0.96)
Burkina Faso	9 (−4, 36)	21 (−10, 83)	132.81	0.22 (−0.08, 0.85)	0.24 (−0.12, 0.94)	0.64 (0.47, 0.81)
Burundi	7 (−3, 34)	11 (−5, 55)	53.20	0.32 (−0.13, 1.49)	0.24 (−0.1, 1.18)	−1.24 (−1.47, −1.01)
Cabo Verde	1 (−1, 6)	4 (−2, 17)	187.70	0.6 (−0.24, 2.63)	0.97 (−0.36, 4.2)	1.18 (0.84, 1.52)
Cambodia	22 (−6, 91)	62 (−21, 287)	185.91	0.5 (−0.15, 2.1)	0.52 (−0.17, 2.36)	0.14 (0.07, 0.22)
Cameroon	15 (−5, 62)	48 (−16, 202)	214.66	0.36 (−0.12, 1.48)	0.41 (−0.13, 1.69)	0.52 (0.44, 0.60)
Canada	443 (−180, 1,550)	628 (−260, 2,151)	41.63	1.36 (−0.55, 4.75)	0.82 (−0.34, 2.82)	−1.48 (−1.64, −1.32)
Central African Republic	5 (−2, 28)	9 (−3, 40)	65.07	0.46 (−0.14, 2.36)	0.38 (−0.11, 1.74)	−0.55 (−0.60, −0.49)
Chad	6 (−2, 24)	17 (−6, 75)	197.47	0.2 (−0.07, 0.86)	0.31 (−0.11, 1.39)	1.66 (1.54, 1.77)
Chile	53 (−19, 220)	109 (−39, 448)	107.08	0.53 (−0.19, 2.21)	0.42 (−0.15, 1.72)	−0.50 (−0.60, −0.39)
China	10,273 (−5,072, 27,729)	29,890 (−15,235, 82,113)	190.97	1.28 (−0.63, 3.47)	1.43 (−0.73, 3.93)	0.41 (0.23, 0.59)
Colombia	147 (−54, 559)	321 (−113, 1,206)	118.56	0.87 (−0.32, 3.3)	0.59 (−0.21, 2.19)	−1.49 (−1.59, −1.38)
Comoros	1 (0, 2)	1 (0, 6)	149.51	0.26 (−0.1, 1.17)	0.27 (−0.1, 1.24)	−0.05 (−0.16, 0.07)
Congo	6 (−2, 26)	13 (−5, 57)	118.29	0.54 (−0.23, 2.39)	0.48 (−0.18, 2.18)	−0.53 (−0.70, −0.37)
Cook Islands	0 (0, 1)	0 (0, 1)	78.61	1.24 (−0.43, 5.35)	0.98 (−0.33, 4.11)	−0.82 (−0.90, −0.74)
Costa Rica	10 (−4, 38)	21 (−7, 81)	113.81	0.57 (−0.21, 2.23)	0.38 (−0.13, 1.47)	−1.33 (−1.52, −1.15)
Croatia	149 (−59, 451)	167 (−71, 504)	12.26	2.37 (−0.94, 7.2)	1.87 (−0.79, 5.63)	−0.55 (−0.66, −0.45)
Cuba	47 (−13, 203)	87 (−26, 385)	83.89	0.46 (−0.13, 1.99)	0.44 (−0.13, 1.94)	0.14 (0.02, 0.25)
Cyprus	1 (−1, 4)	3 (−1, 10)	126.81	0.17 (−0.08, 0.54)	0.13 (−0.06, 0.46)	−0.18 (−0.38, 0.01)
Czechia	655 (−271, 1,921)	629 (−260, 1,921)	−3.88	4.76 (−1.97, 13.99)	2.82 (−1.16, 8.59)	−1.69 (−1.75, −1.63)
Côte d’Ivoire	7 (−2, 33)	19 (−6, 86)	171.22	0.19 (−0.07, 0.88)	0.18 (−0.06, 0.81)	−0.36 (−0.51, −0.22)
Democratic People’s Republic of Korea	200 (−64, 895)	398 (−152, 1,622)	98.92	1.26 (−0.41, 5.62)	1.2 (−0.46, 4.91)	0.08 (−0.05, 0.20)
Democratic Republic of the Congo	50 (−23, 243)	108 (−46, 550)	115.72	0.33 (−0.15, 1.62)	0.3 (−0.13, 1.55)	−0.30 (−0.60, 0)
Denmark	247 (−114, 681)	261 (−113, 723)	5.77	3.11 (−1.44, 8.56)	2.09 (−0.91, 5.78)	−1.15 (−1.24, −1.05)
Djibouti	0 (0, 1)	2 (−1, 9)	515.95	0.25 (−0.09, 1.1)	0.34 (−0.11, 1.57)	0.96 (0.93, 0.99)
Dominica	0 (0, 1)	0 (0, 2)	46.82	0.56 (−0.21, 2.35)	0.58 (−0.2, 2.5)	0.27 (0.15, 0.39)
Dominican Republic	12 (−3, 51)	42 (−13, 184)	246.36	0.34 (−0.1, 1.43)	0.42 (−0.13, 1.85)	1.10 (0.95, 1.26)
Ecuador	24 (−10, 99)	65 (−26, 267)	174.85	0.46 (−0.19, 1.93)	0.41 (−0.16, 1.67)	−0.38 (−0.69, −0.08)
Egypt	18 (−7, 60)	98 (−38, 344)	439.45	0.06 (−0.03, 0.22)	0.16 (−0.06, 0.57)	4.11 (3.56, 4.68)
El Salvador	12 (−5, 53)	28 (−12, 126)	127.88	0.42 (−0.18, 1.81)	0.45 (−0.19, 2.03)	0.05 (−0.09, 0.19)
Equatorial Guinea	1 (0, 3)	3 (−1, 11)	216.12	0.41 (−0.2, 1.67)	0.52 (−0.21, 2.39)	1.07 (0.92, 1.23)
Eritrea	3 (−1, 14)	9 (−3, 39)	161.62	0.28 (−0.12, 1.21)	0.31 (−0.13, 1.36)	0.26 (0.10, 0.42)
Estonia	62 (−26, 188)	49 (−20, 155)	−20.82	2.99 (−1.26, 9.13)	1.8 (−0.74, 5.71)	−1.69 (−1.81, −1.57)
Eswatini	2 (−1, 9)	5 (−1, 22)	146.97	0.7 (−0.22, 3.3)	0.85 (−0.23, 3.86)	1.02 (0.50, 1.54)
Ethiopia	100 (−43, 298)	127 (−53, 389)	26.29	0.5 (−0.22, 1.52)	0.31 (−0.13, 0.96)	−1.84 (−2.08, −1.60)
Fiji	1 (0, 5)	2 (−1, 11)	95.54	0.35 (−0.14, 1.59)	0.32 (−0.13, 1.47)	−0.56 (−0.75, −0.37)
Finland	165 (−68, 498)	209 (−85, 603)	26.25	2.29 (−0.95, 6.9)	1.57 (−0.64, 4.53)	−1.11 (−1.21, −1.01)
France	1,356 (−566, 4,320)	2,025 (−810, 6,351)	49.30	1.7 (−0.71, 5.42)	1.52 (−0.6, 4.74)	−0.07 (−0.25, 0.10)
Gabon	3 (−1, 14)	6 (−2, 26)	87.41	0.52 (−0.16, 2.39)	0.55 (−0.16, 2.46)	0.09 (0.04, 0.14)
Gambia	0 (0, 2)	1 (−1, 6)	193.83	0.13 (−0.05, 0.58)	0.14 (−0.06, 0.62)	0.04 (−0.09, 0.18)
Georgia	112 (−48, 519)	99 (−43, 436)	−11.37	1.74 (−0.75, 8.06)	1.68 (−0.72, 7.35)	1.16 (0.72, 1.60)
Germany	2,253 (−977, 6,768)	2,774 (−1,233, 8,091)	23.13	1.8 (−0.78, 5.4)	1.45 (−0.64, 4.27)	−0.56 (−0.64, −0.48)
Ghana	16 (−6, 57)	48 (−17, 184)	203.79	0.28 (−0.11, 1.01)	0.32 (−0.11, 1.21)	0.71 (0.61, 0.81)
Greece	505 (−247, 1,393)	675 (−322, 1,863)	33.78	3.28 (−1.6, 9.05)	2.88 (−1.37, 7.95)	−0.47 (−0.51, −0.42)
Greenland	3 (−1, 10)	3 (−1, 13)	27.15	8.2 (−3.23, 29.9)	4.91 (−2.05, 18.28)	−1.69 (−1.83, −1.54)
Grenada	0 (0, 1)	0 (0, 2)	37.37	0.37 (−0.12, 1.67)	0.32 (−0.10, 1.33)	−0.22 (−0.64, 0.21)
Guam	1 (0, 4)	2 (−1, 7)	103.61	1.13 (−0.48, 5.5)	0.74 (−0.29, 3.29)	−0.75 (−1.05, −0.46)
Guatemala	16 (−5, 66)	35 (−11, 147)	113.89	0.52 (−0.17, 2.09)	0.32 (−0.11, 1.38)	−2.06 (−2.36, −1.76)
Guinea	9 (−4, 38)	20 (−8, 85)	113.78	0.29 (−0.12, 1.18)	0.36 (−0.15, 1.56)	0.94 (0.83, 1.05)
Guinea-Bissau	1 (0, 7)	3 (−1, 10)	74.89	0.38 (−0.12, 1.69)	0.38 (−0.13, 1.54)	0.31 (0.13, 0.48)
Guyana	1 (0, 3)	1 (0, 6)	64.85	0.2 (−0.08, 0.89)	0.19 (−0.07, 0.85)	0.16 (−0.06, 0.38)
Haiti	13 (−4, 62)	23 (−7, 117)	72.96	0.42 (−0.14, 1.98)	0.34 (−0.11, 1.71)	−0.54 (−0.62, −0.46)
Honduras	12 (−6, 48)	59 (−31, 240)	377.04	0.62 (−0.31, 2.39)	0.97 (−0.5, 3.94)	1.77 (1.60, 1.94)
Hungary	545 (−241, 1,577)	652 (−265, 1,891)	19.58	3.73 (−1.65, 10.81)	3.39 (−1.38, 9.83)	−0.3 (−0.45, −0.16)
Iceland	1 (0, 4)	2 (−1, 6)	64.43	0.39 (−0.17, 1.26)	0.31 (−0.13, 1.00)	−0.49 (−0.69, −0.29)
India	1,070 (−536, 2,905)	3,258 (−1,537, 8,872)	204.49	0.23 (−0.11, 0.62)	0.27 (−0.13, 0.74)	0.51 (0.36, 0.65)
Indonesia	305 (−140, 876)	1,027 (−420, 3,056)	236.26	0.32 (−0.15, 0.93)	0.45 (−0.18, 1.35)	1.04 (0.97, 1.11)
Iran (Islamic Republic of)	131 (−67, 362)	393 (−196, 1,057)	199.61	0.54 (−0.28, 1.51)	0.53 (−0.27, 1.43)	0.27 (0.15, 0.39)
Iraq	41 (−18, 171)	146 (−62, 645)	256.22	0.53 (−0.23, 2.21)	0.67 (−0.28, 2.92)	0.59 (0.43, 0.75)
Ireland	131 (−56, 372)	155 (−63, 453)	18.46	3.11 (−1.33, 8.85)	1.91 (−0.78, 5.6)	−1.28 (−1.42, −1.14)
Israel	53 (−23, 161)	106 (−47, 313)	98.93	1.1 (−0.48, 3.34)	0.84 (−0.37, 2.5)	−0.78 (−0.93, −0.62)
Italy	2,227 (−1,191, 6,232)	2,512 (−1,369, 6,803)	12.78	2.49 (−1.33, 6.98)	1.66 (−0.9, 4.49)	−1.32 (−1.38, −1.27)
Jamaica	9 (−3, 36)	14 (−5, 63)	66.54	0.49 (−0.18, 2.04)	0.46 (−0.18, 2.03)	−0.25 (−0.66, 0.15)
Japan	692 (−315, 1,723)	1,485 (−688, 3,738)	114.50	0.41 (−0.19, 1.02)	0.34 (−0.16, 0.86)	−0.59 (−0.67, −0.51)
Jordan	9 (−4, 27)	37 (−16, 117)	331.62	0.65 (−0.27, 2.06)	0.52 (−0.22, 1.64)	−0.55 (−0.78, −0.32)
Kazakhstan	457 (−154, 1,659)	236 (−86, 878)	−48.34	3.47 (−1.17, 12.58)	1.27 (−0.46, 4.73)	−3.24 (−3.41, −3.06)
Kenya	7 (−3, 19)	32 (−14, 87)	361.21	0.09 (−0.04, 0.24)	0.14 (−0.06, 0.39)	1.79 (1.61, 1.97)
Kiribati	0 (0, 1)	0 (0, 1)	123.71	0.39 (−0.18, 1.72)	0.45 (−0.17, 2.08)	0.47 (0.38, 0.56)
Kuwait	2 (−1, 6)	5 (−2, 15)	157.97	0.33 (−0.15, 1.05)	0.18 (−0.08, 0.59)	−1.43 (−1.80, −1.05)
Kyrgyzstan	60 (−18, 243)	40 (−13, 154)	−34.08	1.96 (−0.6, 7.97)	0.81 (−0.27, 3.12)	−2.47 (−2.79, −2.16)
Lao People’s Democratic Republic	11 (−5, 47)	21 (−8, 85)	88.11	0.54 (−0.23, 2.28)	0.48 (−0.18, 1.92)	−0.44 (−0.50, −0.38)
Latvia	110 (−51, 340)	77 (−36, 239)	−29.88	3.03 (−1.41, 9.4)	1.98 (−0.93, 6.11)	−1.41 (−1.56, −1.25)
Lebanon	22 (−7, 95)	60 (−24, 275)	169.63	1.06 (−0.31, 4.47)	0.99 (−0.39, 4.51)	0.63 (0.27, 0.99)
Lesotho	5 (−3, 22)	13 (−7, 56)	165.11	0.59 (−0.35, 2.63)	1.18 (−0.66, 5.02)	2.85 (2.50, 3.20)
Liberia	3 (−1, 15)	5 (−2, 23)	67.38	0.28 (−0.12, 1.32)	0.27 (−0.09, 1.18)	0.27 (−0.01, 0.54)
Libya	17 (−7, 82)	49 (−21, 226)	187.78	0.95 (−0.4, 4.54)	0.98 (−0.42, 4.48)	0.37 (0.16, 0.59)
Lithuania	74 (−27, 244)	64 (−25, 216)	−13.62	1.62 (−0.58, 5.36)	1.13 (−0.44, 3.84)	−1.26 (−1.38, −1.15)
Luxembourg	21 (−9, 58)	26 (−12, 71)	21.55	3.88 (−1.66, 10.65)	2.41 (−1.12, 6.61)	−1.31 (−1.44, −1.17)
Madagascar	13 (−4, 52)	25 (−8, 108)	97.36	0.25 (−0.08, 1.05)	0.23 (−0.08, 0.99)	−0.26 (−0.39, −0.13)
Malawi	3 (−1, 14)	8 (−3, 32)	128.06	0.09 (−0.04, 0.37)	0.1 (−0.04, 0.43)	0.31 (0.09, 0.53)
Malaysia	22 (−9, 72)	74 (−31, 250)	238.35	0.25 (−0.1, 0.81)	0.27 (−0.11, 0.9)	0.07 (−0.18, 0.31)
Maldives	0 (0, 1)	1 (0, 2)	102.52	0.31 (−0.12, 1.27)	0.17 (−0.05, 0.71)	−2.47 (−2.60, −2.33)
Mali	7 (−3, 35)	17 (−7, 79)	133.33	0.19 (−0.07, 0.92)	0.21 (−0.08, 0.95)	0.51 (0.37, 0.64)
Malta	4 (−2, 18)	7 (−3, 28)	54.13	1.03 (−0.42, 4.11)	0.7 (−0.28, 2.82)	−1.17 (−1.28, −1.07)
Marshall Islands	0 (0, 0)	0 (0, 1)	145.71	0.67 (−0.22, 3.07)	0.79 (−0.23, 3.52)	0.77 (0.65, 0.89)
Mauritania	3 (−1, 11)	6 (−3, 27)	116.67	0.3 (−0.14, 1.15)	0.31 (−0.16, 1.33)	0.18 (−0.08, 0.43)
Mauritius	3 (−1, 12)	5 (−2, 22)	86.19	0.37 (−0.14, 1.64)	0.26 (−0.1, 1.2)	−1.36 (−1.59, −1.13)
Mexico	329 (−162, 836)	512 (−258, 1,299)	55.81	0.83 (−0.41, 2.12)	0.42 (−0.21, 1.06)	−2.57 (−2.69, −2.45)
Micronesia (Federated States of)	0 (0, 2)	1 (0, 3)	67.63	0.8 (−0.31, 3.54)	0.89 (−0.32, 4.12)	0.42 (0.38, 0.46)
Monaco	2 (−1, 6)	3 (−1, 11)	80.80	2.37 (−1.1, 8.72)	2.99 (−1.4, 11.61)	0.93 (0.57, 1.29)
Mongolia	33 (−14, 131)	50 (−19, 196)	52.01	3.16 (−1.31, 12.54)	2.25 (−0.87, 8.83)	−1.56 (−1.75, −1.37)
Montenegro	11 (−5, 42)	19 (−9, 71)	75.35	1.72 (−0.82, 6.68)	1.93 (−0.92, 7.22)	0.54 (0.40, 0.68)
Morocco	106 (−48, 394)	281 (−131, 1,010)	163.85	0.76 (−0.34, 2.83)	0.82 (−0.38, 2.97)	0.32 (0.15, 0.49)
Mozambique	11 (−3, 44)	25 (−8, 107)	141.33	0.2 (−0.06, 0.83)	0.25 (−0.08, 1.09)	1.35 (1.19, 1.51)
Myanmar	119 (−45, 528)	200 (−81, 881)	68.24	0.52 (−0.2, 2.29)	0.42 (−0.17, 1.86)	−0.79 (−0.84, −0.74)
Namibia	1 (−1, 7)	3 (−1, 14)	140.31	0.21 (−0.09, 1.01)	0.24 (−0.09, 1.05)	0.32 (0.06, 0.58)
Nauru	0 (0, 0)	0 (0, 0)	16.04	1.22 (−0.4, 5.93)	1.15 (−0.32, 5.38)	−0.14 (−0.16, −0.12)
Nepal	29 (−11, 117)	72 (−28, 278)	147.63	0.31 (−0.12, 1.25)	0.32 (−0.12, 1.21)	0.13 (−0.13, 0.39)
Netherlands	202 (−88, 660)	253 (−110, 816)	25.30	1 (−0.44, 3.28)	0.7 (−0.3, 2.25)	−1.05 (−1.15, −0.96)
New Zealand	42 (−14, 165)	60 (−22, 223)	41.59	1.06 (−0.35, 4.12)	0.69 (−0.25, 2.59)	−1.38 (−1.46, −1.29)
Nicaragua	4 (−1, 16)	11 (−4, 46)	182.89	0.27 (−0.1, 1.11)	0.24 (−0.08, 0.98)	−0.29 (−0.51, −0.06)
Niger	5 (−2, 21)	14 (−5, 59)	189.40	0.19 (−0.07, 0.8)	0.19 (−0.06, 0.8)	0.41 (0.23, 0.60)
Nigeria	26 (−13, 71)	57 (−29, 154)	122.04	0.06 (−0.03, 0.17)	0.07 (−0.03, 0.18)	0.65 (0.55, 0.75)
Niue	0 (0, 0)	0 (0, 0)	15.55	0.77 (−0.25, 3.48)	0.94 (−0.3, 4.08)	0.66 (0.57, 0.75)
North Macedonia	52 (−28, 157)	96 (−47, 295)	85.24	2.7 (−1.44, 8.08)	2.84 (−1.39, 8.71)	0.24 (−0.06, 0.55)
Northern Mariana Islands	0 (0, 1)	1 (0, 2)	150.20	1.45 (−0.44, 6.7)	1.13 (−0.35, 5.19)	−0.87 (−0.94, −0.81)
Norway	111 (−48, 302)	169 (−76, 458)	52.54	1.65 (−0.72, 4.5)	1.62 (−0.72, 4.39)	−0.02 (−0.21, 0.17)
Oman	1 (0, 5)	3 (−1, 10)	107.09	0.19 (−0.08, 0.79)	0.14 (−0.05, 0.55)	−0.63 (−0.9, −0.36)
Pakistan	315 (−123, 1,013)	757 (−292, 2,484)	140.53	0.57 (−0.22, 1.85)	0.64 (−0.25, 2.09)	0.10 (−0.15, 0.36)
Palau	0 (0, 1)	0 (0, 1)	110.22	1.49 (−0.57, 6.82)	1.44 (−0.53, 6.71)	0.04 (−0.02, 0.10)
Palestine	8 (−3, 31)	22 (−9, 77)	156.91	1.02 (−0.41, 3.74)	0.91 (−0.38, 3.22)	−0.42 (−0.64, −0.20)
Panama	9 (−3, 44)	17 (−6, 84)	84.36	0.65 (−0.21, 3.03)	0.39 (−0.13, 1.89)	−1.65 (−1.82, −1.48)
Papua New Guinea	9 (−3, 42)	28 (−11, 116)	205.15	0.56 (−0.19, 2.6)	0.62 (−0.23, 2.55)	0.45 (0.39, 0.51)
Paraguay	7 (−3, 23)	28 (−11, 103)	319.72	0.31 (−0.13, 1.08)	0.5 (−0.19, 1.82)	1.62 (1.35, 1.89)
Peru	64 (−20, 304)	137 (−48, 657)	114.20	0.55 (−0.17, 2.62)	0.41 (−0.14, 1.98)	−1.21 (−1.56, −0.85)
Philippines	108 (−55, 283)	288 (−142, 742)	167.33	0.39 (−0.2, 1.02)	0.36 (−0.18, 0.92)	−0.5 (−0.65, −0.34)
Poland	789 (−349, 2,201)	1,204 (−552, 3,279)	52.55	1.78 (−0.79, 4.97)	1.63 (−0.75, 4.45)	−0.38 (−0.54, −0.23)
Portugal	186 (−75, 543)	309 (−133, 939)	65.98	1.33 (−0.54, 3.89)	1.29 (−0.55, 3.91)	0.05 (−0.08, 0.17)
Puerto Rico	18 (−7, 86)	20 (−8, 97)	10.92	0.51 (−0.18, 2.37)	0.28 (−0.1, 1.32)	−1.85 (−2.03, −1.67)
Qatar	1 (0, 3)	3 (−1, 13)	326.56	0.74 (−0.28, 3.28)	0.37 (−0.14, 1.64)	−2.34 (−2.93, −1.75)
Republic of Korea	442 (−185, 1,386)	1,275 (−621, 4,144)	188.14	1.52 (−0.64, 4.77)	1.34 (−0.66, 4.37)	−0.82 (−1.17, −0.48)
Republic of Moldova	84 (−28, 322)	67 (−22, 253)	−20.60	1.84 (−0.62, 7.06)	1.1 (−0.37, 4.16)	−1.08 (−1.42, −0.74)
Romania	332 (−156, 1,079)	514 (−231, 1,680)	54.53	1.15 (−0.54, 3.72)	1.44 (−0.64, 4.7)	0.62 (0.49, 0.74)
Russian	4,791 (−2,353, 12,365)	3,985 (−1,972, 10,415)	−16.83	2.56 (−1.26, 6.61)	1.63 (−0.81, 4.26)	−1.64 (−1.77, −1.50)
Rwanda	11 (−4, 41)	20 (−6, 85)	87.02	0.38 (−0.13, 1.47)	0.34 (−0.1, 1.38)	−1.14 (−1.46, −0.82)
Saint Kitts and Nevis	0 (0, 1)	0 (0, 1)	55.05	0.32 (−0.14, 1.43)	0.28 (−0.12, 1.28)	−0.01 (−0.19, 0.16)
Saint Lucia	0 (0, 2)	1 (0, 3)	109.36	0.43 (−0.15, 1.93)	0.32 (−0.11, 1.44)	−1.04 (−1.23, −0.85)
Saint Vincent and the Grenadines	0 (0, 1)	0 (0, 1)	74.19	0.28 (−0.09, 1.17)	0.25 (−0.08, 1.05)	−0.13 (−0.28, 0.02)
Samoa	0 (0, 1)	0 (0, 2)	64.64	0.26 (−0.11, 1.18)	0.25 (−0.12, 1.09)	−0.07 (−0.14, 0)
San Marino	1 (0, 2)	1 (0, 2)	2.09	1.79 (−0.71, 6.58)	0.88 (−0.33, 3.31)	−1.33 (−1.69, −0.98)
Sao Tome and Principe	0 (0, 2)	1 (0, 4)	114.99	0.65 (−0.3, 2.54)	0.88 (−0.4, 3.49)	1.24 (1.16, 1.32)
Saudi Arabia	6 (−3, 20)	20 (−10, 58)	226.12	0.11 (−0.05, 0.35)	0.11 (−0.06, 0.32)	−0.04 (−0.24, 0.17)
Senegal	10 (−4, 46)	27 (−11, 123)	167.09	0.32 (−0.13, 1.46)	0.36 (−0.15, 1.7)	0.63 (0.41, 0.85)
Serbia	284 (−132, 1,006)	399 (−162, 1,394)	40.48	2.47 (−1.15, 8.78)	2.44 (−0.99, 8.55)	0.09 (−0.05, 0.23)
Seychelles	0 (0, 1)	0 (0, 2)	44.35	0.43 (−0.19, 1.76)	0.31 (−0.13, 1.37)	−1.08 (−1.25, −0.91)
Sierra Leone	6 (−2, 23)	10 (−4, 42)	80.69	0.28 (−0.12, 1.14)	0.28 (−0.11, 1.17)	0.48 (0.25, 0.71)
Singapore	7 (−3, 30)	13 (−6, 58)	90.13	0.33 (−0.14, 1.44)	0.16 (−0.07, 0.69)	−2.41 (−2.54, −2.27)
Slovakia	220 (−95, 644)	216 (−102, 637)	−1.80	3.65 (−1.58, 10.69)	2.23 (−1.06, 6.59)	−1.5 (−1.57, −1.44)
Slovenia	69 (−30, 207)	97 (−41, 293)	41.02	2.75 (−1.19, 8.29)	2.15 (−0.9, 6.5)	−0.9 (−1.03, −0.78)
Solomon Islands	1 (0, 4)	2 (−1, 11)	164.35	0.64 (−0.2, 3.04)	0.65 (−0.19, 3.08)	0.06 (−0.11, 0.23)
Somalia	5 (−2, 21)	11 (−5, 47)	133.56	0.19 (−0.1, 0.87)	0.17 (−0.08, 0.78)	−0.22 (−0.28, −0.15)
South Africa	180 (−87, 522)	433 (−198, 1,327)	141.02	0.87 (−0.42, 2.51)	0.94 (−0.43, 2.89)	0.15 (−0.15, 0.44)
South Sudan	6 (−3, 27)	9 (−4, 47)	50.40	0.24 (−0.1, 1.06)	0.25 (−0.1, 1.26)	0.16 (0.07, 0.26)
Spain	1,178 (−533, 3,384)	1,656 (−730, 4,644)	40.58	2.16 (−0.98, 6.2)	1.74 (−0.76, 4.89)	−0.59 (−0.71, −0.48)
Sri Lanka	20 (−8, 87)	44 (−15, 185)	119.10	0.19 (−0.08, 0.84)	0.16 (−0.06, 0.68)	−0.32 (−0.57, −0.08)
Sudan	20 (−8, 85)	48 (−20, 215)	139.61	0.22 (−0.09, 0.94)	0.26 (−0.11, 1.16)	0.66 (0.58, 0.74)
Suriname	1 (0, 4)	2 (−1, 10)	135.39	0.39 (−0.11, 1.71)	0.36 (−0.11, 1.59)	0.09 (−0.14, 0.32)
Sweden	200 (−77, 697)	262 (−101, 946)	30.61	1.35 (−0.52, 4.7)	1.12 (−0.44, 4.07)	−0.32 (−0.5, −0.15)
Switzerland	201 (−95, 589)	256 (−121, 744)	27.11	1.98 (−0.94, 5.78)	1.39 (−0.66, 4.03)	−0.88 (−0.98, −0.77)
Syrian Arab Republic	23 (−10, 76)	55 (−20, 182)	139.04	0.45 (−0.2, 1.49)	0.44 (−0.16, 1.44)	−0.26 (−0.39, −0.12)
Taiwan (Province of China)	44 (−20, 135)	123 (−57, 382)	181.54	0.28 (−0.13, 0.86)	0.29 (−0.13, 0.89)	−0.03 (−0.34, 0.28)
Tajikistan	28 (−13, 121)	28 (−13, 113)	0.22	1.01 (−0.48, 4.29)	0.48 (−0.21, 1.9)	−2.18 (−2.45, −1.90)
Thailand	199 (−81, 698)	472 (−232, 1,433)	137.40	0.59 (−0.24, 2.06)	0.43 (−0.21, 1.32)	−1.55 (−1.75, −1.35)
Timor-Leste	1 (0, 3)	3 (−1, 12)	262.44	0.29 (−0.14, 1.22)	0.32 (−0.14, 1.41)	0.37 (0.20, 0.54)
Togo	4 (−1, 15)	13 (−5, 54)	254.85	0.31 (−0.1, 1.24)	0.36 (−0.13, 1.52)	0.49 (0.44, 0.55)
Tokelau	0 (0, 0)	0 (0, 0)	1.77	1.45 (−0.49, 6.1)	1.54 (−0.58, 6.53)	0.28 (0.24, 0.31)
Tonga	0 (0, 2)	1 (0, 3)	51.11	0.88 (−0.4, 3.94)	0.90 (−0.38, 3.79)	0.01 (−0.17, 0.19)
Trinidad and Tobago	3 (−1, 12)	5 (−1, 25)	96.01	0.33 (−0.09, 1.52)	0.27 (−0.07, 1.26)	−0.63 (−0.73, −0.53)
Tunisia	50 (−21, 157)	122 (−49, 381)	145.69	1 (−0.43, 3.17)	0.9 (−0.36, 2.83)	−0.61 (−0.74, −0.48)
Turkey	675 (−262, 2,128)	1,249 (−537, 3,694)	85.04	0.87 (−0.33, 3.58)	0.46 (−0.17, 1.83)	−1.24 (−1.49, −0.99)
Turkmenistan	18 (−7, 72)	20 (−7, 78)	12.01	0.75 (−0.3, 3.46)	0.85 (−0.36, 3.8)	−1.99 (−2.42, −1.56)
Tuvalu	0 (0, 0)	0 (0, 0)	81.88	1.93 (−0.75, 6.07)	1.32 (−0.57, 3.91)	0.38 (0.33, 0.43)
Uganda	14 (−5, 57)	35 (−13, 155)	157.65	0.22 (−0.09, 0.9)	0.25 (−0.09, 1.11)	−0.13 (−0.40, 0.15)
Ukraine	1,655 (−664, 5,827)	805 (−296, 2,648)	−51.34	2.27 (−0.91, 8.01)	1.05 (−0.38, 3.45)	−2.80 (−3.00, −2.61)
United Arab Emirates	2 (−1, 10)	12 (−5, 48)	385.23	0.59 (−0.23, 2.27)	0.42 (−0.17, 1.7)	0.22 (−0.34, 0.78)
United Kingdom	2,353 (−1,089, 5,989)	2,154 (−1,015, 5,485)	−8.45	2.55 (−1.18, 6.5)	1.56 (−0.73, 3.97)	−1.44 (−1.52, −1.36)
United Republic of Tanzania	27 (−10, 114)	67 (−28, 292)	147.11	0.25 (−0.09, 1.05)	0.27 (−0.11, 1.18)	0.12 (0.07, 0.17)
United States of America	7,218 (−3,380, 18,770)	7,962 (−3,751, 20,761)	10.31	2.27 (−1.07, 5.91)	1.31 (−0.62, 3.42)	−1.92 (−2.11, −1.72)
United States Virgin Islands	0 (0, 2)	1 (0, 3)	57.65	0.49 (−0.16, 2.18)	0.35 (−0.11, 1.59)	−0.92 (−1.14, −0.70)
Uruguay	51 (−14, 241)	57 (−15, 271)	11.43	1.3 (−0.35, 6.21)	1.04 (−0.28, 5)	−0.74 (−0.83, −0.66)
Uzbekistan	115 (−44, 466)	111 (−46, 468)	−3.86	0.98 (−0.37, 3.96)	0.41 (−0.17, 1.71)	−2.72 (−3.03, −2.40)
Vanuatu	0 (0, 1)	1 (0, 4)	191.17	0.59 (−0.29, 2.43)	0.61 (−0.3, 2.66)	0.12 (0.07, 0.17)
Venezuela (Bolivarian Republic of)	84 (−31, 360)	245 (−88, 1,073)	191.24	0.88 (−0.33, 3.79)	0.82 (−0.3, 3.6)	−0.19 (−0.32, −0.07)
Viet Nam	170 (−62, 692)	471 (−189, 2,087)	176.52	0.42 (−0.15, 1.7)	0.47 (−0.19, 2.08)	0.22 (0.14, 0.30)
Yemen	17 (−7, 74)	51 (−23, 217)	199.30	0.35 (−0.14, 1.48)	0.38 (−0.17, 1.62)	0.49 (0.39, 0.60)
Zambia	9 (−4, 38)	28 (−11, 125)	229.10	0.31 (−0.14, 1.34)	0.41 (−0.16, 1.8)	0.79 (0.69, 0.90)
Zimbabwe	21 (−8, 92)	41 (−16, 191)	96.49	0.53 (−0.2, 2.35)	0.61 (−0.23, 2.86)	0.58 (0.25, 0.91)

UI, uncertainty intervals; EAPC, estimated annual percentage change.

### Burden of LC based on sex and age

**Figure 2:**
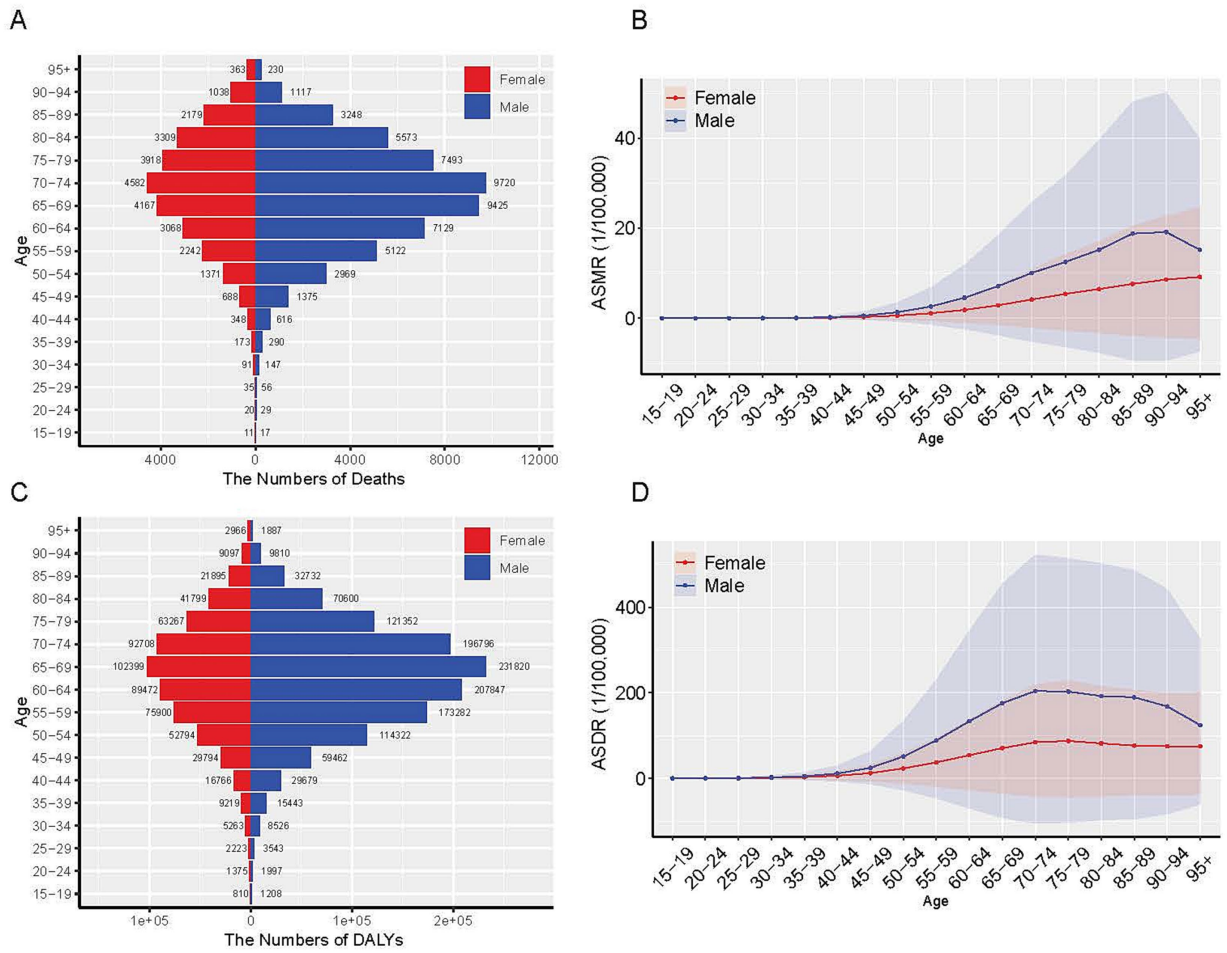
The global disease burden of LC attributed to residential radon exposure by age and sex in 2021. **(A)** The number of deaths; **(B)** ASMR; **(C)** The number of DALYs; **(D)** ASDR. ASMR, age-standardized mortality rate; DALYs, disability-adjusted life years; ASDR, age-standardized DALYs rate.

In 2021, both males and females exhibited rapid increases in deaths and DALYs number around age 60. Mortality peaked in the 70–74 age group, while DALYs reached their maximum in the 65–69 age group (Figure 2). ASMR exhibited distinct sex-specific patterns. Females demonstrated a progressive increase with advancing age, whereas males exhibited an initial increase peaking at 90–94 years, followed by a subsequent decline. Both female and male exhibited gradual increases in ASDR, reaching maximum values in the 70–74 age group for males and the 75–79 age group for females (Figure 2). Notably, males consistently displayed higher disease burden than females across all age groups. Collectively, these findings indicated significantly greater health impacts associated with residential radon exposure among male populations in 2021.

### Burden of LC based on SDI

**Figure 3:**
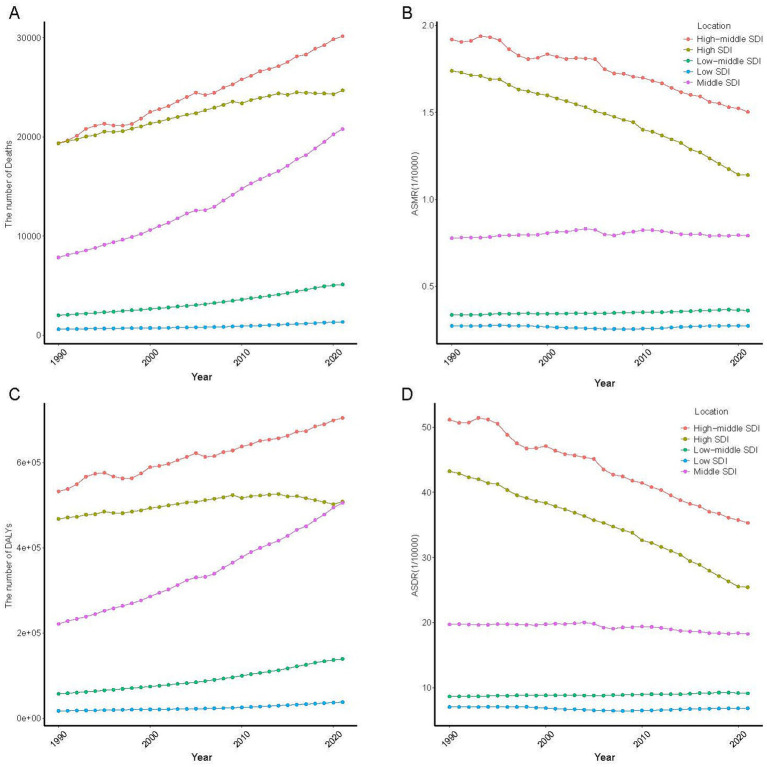
Temporal trend of LC attributed to residential radon exposure in 5 SDI region. **(A)** The number of deaths; **(B)** ASMR; **(C)** The number of DALYs; **(D)** ASDR. ASMR, age-standardized mortality rate; DALYs, disability-adjusted life years; ASDR, age-standardized DALYs rate.

In 2021, the high-middle SDI region accounted for the highest absolute deaths and DALYs numbers of LC attributable to residential radon exposure, representing approximately 40% of the global total (Supplementary Table S3, S4). Over the past three decades, all five SDI regions exhibited progressive increases in deaths and DALYs numbers. However, ASRs demonstrated declining trends in high SDI and high-middle SDI regions since 1990, while remaining relatively stable in the other three SDI regions (Figure 3).

**Figure 4:**
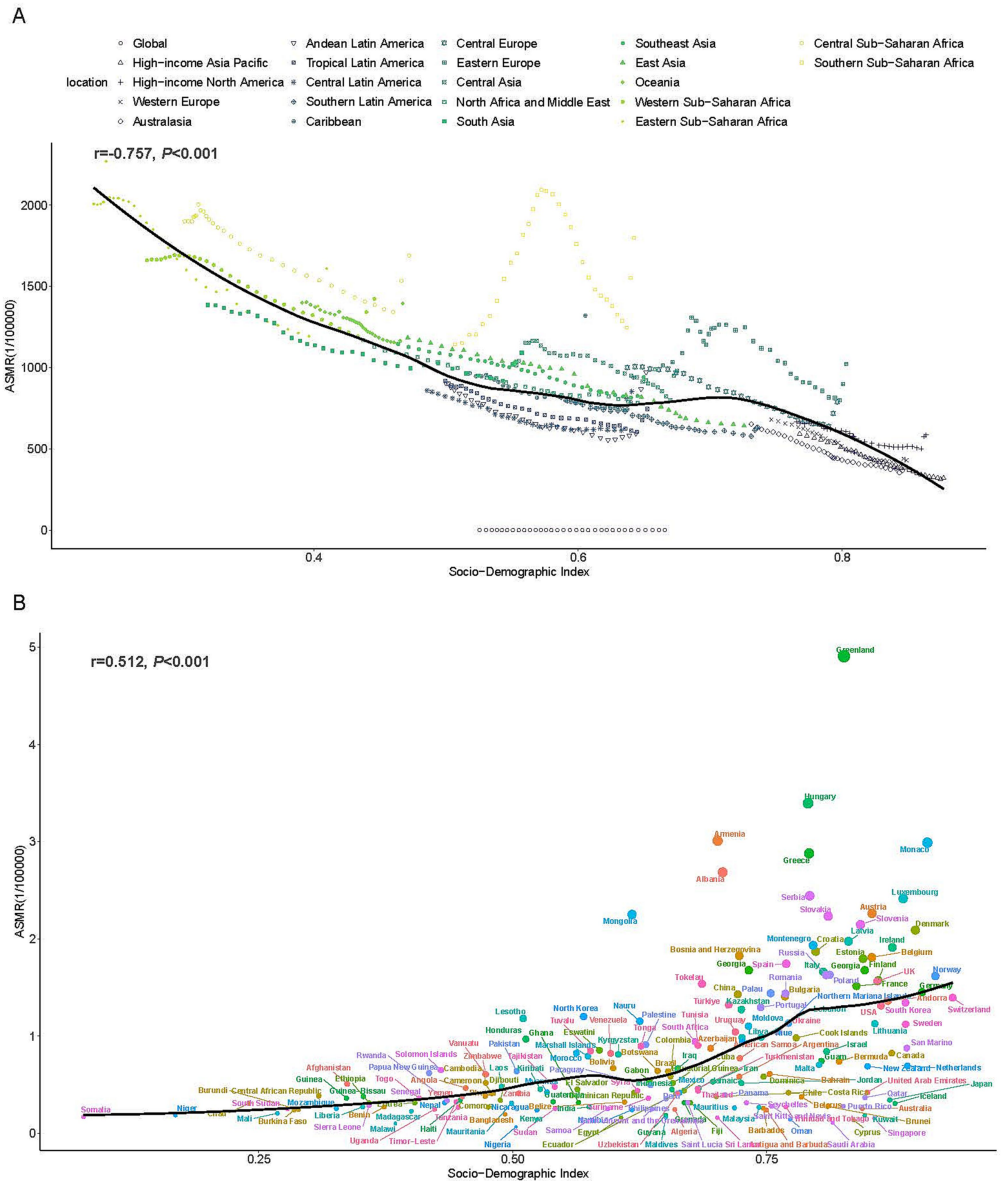
The associations between the SDI and ASMR of LC attributed to residential radon exposure across. **(A)** The associations between the SDI and ASMR in 21 regions and global according from 1990 to 2021. **(B)** The associations between the SDI and ASMR in 204 countries in 2021. The black line was the expected values based on the SDI and disease rates. SDI, socio-demographic index; ASMR, age-standardized mortality rate.

Significant negative correlations were identified between SDI and both ASMR (*r* = −0.757, *p* < 0.001) and ASDR (*r* = −0.824, *p* < 0.001) for residential radon-induced LC (Figure 4 and Supplementary Figure S2). With the exception of Southern Sub-Saharan Africa, all regions exhibited negative correlations between SDI levels and temporal trends in ASRs from 1990 to 2021. National-level analyses revealed contrasting patterns in 2021, with positive correlations observed between SDI and both ASMR (*r* = 0.512, *p* < 0.01) and ASDR (*r* = 0.478, *p* < 0.01) across 204 countries (Figure 4 and Supplementary Figure S2).

## Discussion

In this study, we conducted an in-depth exploration on the burden of LC attributed to residential radon exposure globally and regionally from 1990 to 2021. The findings elucidated disease burden trends of LC attributed to residential radon exposure according to geographic location, SDI, age and sex, which will assist decision-makers in evaluating the overall burden of LC due to residential radon exposure and facilitating equitable distribution of public health resources.

Globally, approximately 80,000 deaths were attributed to residential radon exposure in 2021, signifying a 66.87% increase since 1990. This upward trajectory underscores radon exposure as a significant and escalating public health concern, particularly in rapidly developing regions. The increase may correlate with advancements in medical diagnostics and an aging population, both contributing to rising LC incidence and mortality (15). Residential radon accounts for 4.1% of total LC mortality risk factors, with its attributable death burden continuing to rise (16). Notably, ASMR (EAPC: −0.26) and ASDR (EAPC: −0.65) for radon-induced LC have declined. This reduction aligns with two key factors. Firstly, global tobacco control initiatives have substantially decreased smoking rates, thereby mitigating the synergistic carcinogenic effects between smoking and radon exposure (17, 18). Secondly, governmental residential radon mitigation policies instituted since radon’s identification as a carcinogen have effectively reduced indoor radon concentrations (19, 20). Despite population growth and aging, the overall global burden of radon-related LC has diminished, illustrating the partial success of current mitigation strategies. This success highlights the necessity for differentiated prevention approaches, including implementing structural radon reduction measures in developing regions and enhancing early detection programs for aging populations (21).

The disease burden of residential radon-induced LC exhibited distinct regional and national variations. East and South Asia have experienced substantial increases in the disease burden, with East Asia accounting for one-third of global residential radon-attributable LC deaths, consistent with its geological features prone to radon and urbanization-driven housing density (2). In contrast, North America and Europe have demonstrated significant reductions in disease burden of residential radon-induced LC compared to 1990, likely due to the implementation of radon control strategies that have lowered indoor radon concentrations. Following national surveys assessing indoor radon concentrations and accumulated experience from remediation initiatives, European countries collectively established National Radon Action Plans (NRAPs) (20). In North America, such as Canada, a residential radon reduction strategy was implemented in 2007, effectively decreasing radon concentrations in residential settings (22). This research also identified that in several mid and high latitude countries, the disease burden of radon-induced LC was particularly pronounced, including countries like Greenland, Russia, Mongolia, Denmark, and Ireland. These regions have a dry and cold climate in winter, and indoor environments are usually enclosed with limited air circulation, making residents more susceptible to exposure to radon (23). Interestingly, in 2021, China, the United States, and Russia had the highest number of deaths and DALYs from LC caused by residential radon exposure. This trend may be attributed to recent environmental protection requirements in the context of economic development, leading to an increase in new building materials that elevate indoor radon concentrations and, consequently, residents’ exposure (24–26).

The research indicated that the disease burden of LC attributable to residential radon exposure was predominantly concentrated in the high-middle SDI region, accounting for 40% of the global total. Most countries within this region are situated in mid or high latitudes, making them more susceptible to residential radon exposure (23). As SDI increases, ASRs trends in most regions exhibit a decline. Higher SDI is associated with improved medical services and greater awareness of radon control, contributing to the primary and secondary prevention of LC, which in turn leads to reductions in ASRs (16). Interestingly, ASRs for radon-induced LC in 2019 demonstrated a positive correlation with SDI in low and middle SDI areas (27), which contrasts with previous findings. The GBD 2021 data introduced a novel method called BPRF that conservatively evaluates the correlation between risk factors and outcomes based on evidence consistency while accounting for heterogeneity among various studies, thereby enhancing the reliability of the results. Furthermore, GBD 2021 systematically adjusted the pathways through which risk factors indirectly affect health outcomes through intermediate variables (13). Notably, national-level analyses revealed a positive correlation between SDI and ASRs. This potentially attributed to accelerated urbanization and industrialization that have transformed lifestyle patterns, resulting in increased indoor activity.

The findings of this study indicated that the male exhibit higher mortality rates, DALYs and ASRs due to residential radon exposure compared to the female, highlighting gender disparities in the burden of LC associated with radon exposure. Males tend to have higher tobacco consumption rates, and research indicates that the majority of LC deaths attributed to radon occur among smokers or former smokers (28). Additionally, occupational exposure to radon may also contribute to LC incidence. Evidence from studies on uranium miners in Germany suggested that even decades after mine closure, a significant proportion of LC deaths remain related to occupational radon exposure, predominantly affecting males. These factors elucidate the higher burden of radon-induced LC among men (29). These reasons can explain why men have a higher burden of radon induced LC.

The study further revealed that the mortality and DALYs number associated with LC increase with age, peaking at 65–74 years. Older individuals often present with various chronic conditions that compromise immunity, increasing their susceptibility to LC due to residential radon exposure. Moreover, radon-induced LC exhibits latent cumulative exposure effects, with disease manifestation occurring only after prolonged exposure (30). The older adult in the world, especially in underdeveloped areas, are often accompanied by high comorbidity and multiple incidence rate (31). The aging global population, particularly in underdeveloped regions, often faces high comorbidity rates and multiple incidences, thereby imposing significant burdens on healthcare and social systems. An unhealthy living environment exacerbates the consumption of public health resources among the older adult. Consequently, local authorities should prioritize interventions for aging male smokers with a history of occupational radon exposure, create a suitable living environment for the older adult, and improve access to healthcare.

There are a few limitations to this study. Firstly, because of the nature of the data, we were unable to examine the incidence and prevalence of LC caused by residential radon exposure. Secondly, exposure estimates rely on regional radon maps that may not capture micro-geographical variations. Thirdly, the data from the GBD study relied on mathematical models, which means that the results of this study may be distorted.

## Conclusion

Residential radon remains a critical yet modifiable LC risk factor. Targeted mitigation in high-risk regions, coupled with enhanced screening for aging populations, is imperative to reduce the global burden. Policymakers must integrate radon control into public health frameworks, prioritizing regions with low SDI and urbanization trends.

## Data Availability

The original contributions presented in the study are included in the article/Supplementary material, further inquiries can be directed to the corresponding author.

## Footnotes

^1^ https://ghdx.healthdata.org/gbd-2021

^2^ https://ghdx.healthdata.org/record/global-burden-disease-study-2021-gbd-2021-socio-demographic-index-sdi-1950%E2%80%932021
