# Supplementary material for: Epidemiological trends of lung cancer attributed to residential radon exposure at global, regional, and national level: a trend analysis study from 1990 to 2021
Source: Front Public Health. 2025 May 2;13:1593415. doi: 10.3389/fpubh.2025.1593415 (PMC12081354; doi:10.3389/fpubh.2025.1593415)
Supplement: Supplementary file 1 [file Data_Sheet_1.DOCX]

Supplementary Table S1 The DALYs number and rate of lung cancer attributed to residential radon exposure in 1990 and 2021 across 21 regions and global, and the trends from 1990 to 2021

| Region | All ages DALYs number | | | Age-standard DALYs rate per 100000 population | | |
| --- | --- | --- | --- | --- | --- | --- |
|  | 1990(95% UI) | 2021(95% UI) | percentage change,% | 1990(95% UI) | 2021(95% UI) | EAPC(95% CI) |
| Global | 1298471(-627033, 3301309) | 1898051(-968774, 4852214) | 46.18 | 31.51(-15.22, 80.26) | 21.73(-11.08, 55.55) | -1.23(-1.28, -1.18) |
| Andean Latin America | 2926(-1373, 10266) | 6146(-2911, 21055) | 110.05 | 13.56(-6.36, 47.48） | 10.25(-4.86, 35.03) | -1.05(-1.25, -0.85) |
| Australasia | 2761(-1255, 9230) | 3675(-1750, 11739) | 33.08 | 11.80(-5.36, 39.26) | 7.15(-3.41, 22.75) | -1.63(-1.68, -1.57) |
| Caribbean | 2803(-1003, 9306) | 5032(-1868, 15640) | 79.50 | 10.61(-3.79, 35.25) | 9.34(-3.47, 28.99) | -0.24(-0.3, -0.17) |
| Central Asia | 30355(-13151, 88357) | 22431(-9729, 62111) | -26.11 | 59.89(-25.90, 174.63) | 25.29(-11.00, 70.34) | -2.59(-2.68, -2.51) |
| Central Europe | 95610(-45341, 246401) | 104944(-52130, 268606) | 9.76 | 62.59(-29.69, 161.36) | 49.76(-24.83, 127.28) | -0.74(-0.85, -0.63) |
| Central Latin America | 16244(-7172, 42380) | 29744(-12405, 81189) | 83.11 | 18.60(-8.27, 48.61) | 11.71(-4.89, 31.91) | -1.72(-1.79, -1.65) |
| Central Sub-Saharan Africa | 2424(-1057, 9164) | 5614(-3010, 22218) | 131.60 | 9.72(-4.21, 36.84) | 9.02(-4.78, 35.66) | -0.24(-0.45, -0.02) |
| East Asia | 294217(-144773, 793779) | 709297(-362701, 1952605) | 141.08 | 31.45(-15.40, 84.78) | 31.79(-16.20, 87.47) | 0.05(-0.11, 0.2) |
| Eastern Europe | 196241(-94383, 507533) | 129378(-63687, 333978) | -34.07 | 68.08(-32.72, 176.20) | 37.29(-18.36, 96.26) | -2.2(-2.33, -2.06) |
| Eastern Sub-Saharan Africa | 6226(-2576, 16708） | 11575(-4894, 30816) | 85.90 | 7.57(-3.13, 20.27) | 6.26(-2.65, 16.68) | -0.79(-0.91, -0.67) |
| High-income Asia Pacific | 27970(-13958, 75692) | 49060(-25643, 137103) | 75.40 | 13.60(-6.79, 36.74) | 11.19(-5.80, 31.43) | -0.77(-0.89, -0.65) |
| High-income North America | 183727(-85820, 480355) | 177766(-84774, 452164) | -3.24 | 54.98(-25.70, 143.68) | 27.34(-13.02, 69.43) | -2.37(-2.54, -2.19) |
| North Africa and Middle East | 34338(-14480, 100885) | 72378(-32625, 197802) | 110.78 | 18.72(-7.86, 55.02) | 15.16(-6.82, 41.65) | -0.62(-0.75, -0.48) |
| Oceania | 445(-184, 1543) | 1163(-521, 4325) | 161.67 | 13.89(-5.72, 47.60) | 14.33(-6.33, 52.89) | 0.16(0.08, 0.23) |
| South Asia | 45408(-21628, 115040) | 121722(-57503, 315762) | 168.06 | 7.09(-3.37, 18.17) | 7.69(-3.63, 19.93) | 0.16(0.07, 0.25) |
| Southeast Asia | 27483(-13091, 79840) | 70714(-31600, 199308) | 157.30 | 9.99(-4.78, 29.04) | 10.15(-4.54, 28.64) | -0.15(-0.23, -0.06) |
| Southern Latin America | 10081(-4046, 34834) | 11387(-4632, 38880) | 12.95 | 21.46(-8.61, 74.16) | 13.29(-5.41, 45.48) | -1.41(-1.51, -1.32) |
| Southern Sub-Saharan Africa | 6206(-3083, 17930) | 13962(-6379, 40363) | 124.98 | 21.10(-10.51, 59.73) | 22.498(-10.30, 65.29) | 0.12(-0.18, 0.42) |
| Tropical Latin America | 17697(-8147, 48518) | 39178(-18160, 110140) | 121.38 | 18.13(-8.37, 49.67) | 14.99(-6.95, 42.14) | -0.65(-0.73, -0.56) |
| Western Europe | 291730(-141799, 771354) | 303697(-141142, 794420) | 4.10 | 53.22(-25.88, 141.17) | 35.96(-16.63, 94.1) | -1.13(-1.21, -1.05) |
| Western Sub-Saharan Africa | 3578(-1598, 9273) | 9188(-4046, 23598) | 156.82 | 3.87(-1.73, 10.01) | 4.41(-1.97, 11.38) | 0.62(0.55, 0.68) |

UI：uncertainty intervals；EAPC: estimated annual percentage change

Supplementary Table S2 The DALYs number and rate of lung cancer attributed to residential radon exposure in 1990 and 2021 across 204 countries, and the trends from 1990 to 2021

| Location | All ages DALYs number | | | Age-standard DALYs rate per 100000 population | | |
| --- | --- | --- | --- | --- | --- | --- |
|  | 1990(95% UI) | 2021(95% UI) | percentage change,% | 1990(95% UI) | 2021(95% UI) | EAPC(95% CI) |
| Afghanistan | 863(-299, 3685) | 1550(-615, 6418) | 79.62 | 11.68(-4.13, 49.81) | 12.95(-5.05, 52.59) | 0.49(0.37, 0.61) |
| Albania | 1746(-945, 4822) | 2755(-1504, 8086) | 57.80 | 80.06(-43.29, 221.8) | 63.38(-34.48, 186.12) | -0.52(-0.75, -0.28) |
| Algeria | 873(-349, 3403) | 2052(-772, 7941) | 135.16 | 6.94(-2.78, 27.18) | 5.55(-2.07, 21.38) | -0.60(-0.77, -0.43) |
| American Samoa | 5(-2, 21) | 9(-3, 38) | 87.15 | 20.15(-6.79, 86.07) | 18.31(-6.42, 75.08) | -0.22(-0.28, -0.16) |
| Andorra | 37(-14, 144) | 48(-20, 187) | 31.36 | 62.1(-23.48, 243.51) | 31.71(-13.4, 123.38) | -1.85(-2.10, -1.60) |
| Angola | 563(-223, 2362) | 1575(-611, 7294) | 179.80 | 12.77(-5.11, 53.71) | 11.75(-4.62, 54.71) | -0.25(-0.39, -0.10) |
| Antigua and Barbuda | 3(-1, 15) | 6(-2, 25) | 76.04 | 6.81(-2.25, 28.69) | 5.52(-1.77, 22.41) | -0.45(-0.69, -0.20) |
| Argentina | 7415(-2973, 29430) | 7690(-3089, 29262) | 3.72 | 22.68(-9.09, 90.11) | 14.05(-5.64, 53.45) | -1.40(-1.54, -1.26) |
| Armenia | 3448(-1731, 11370) | 3246(-1610, 10364) | -5.84 | 113.31(-56.8, 373.64) | 74.16(-36.76, 236.88) | -1.22(-1.46, -0.98) |
| Australia | 1769(-731, 6357) | 2404(-1024, 8642) | 35.89 | 9.06(-3.75, 32.62) | 5.54(-2.35, 19.92) | -1.61(-1.65, -1.56) |
| Austria | 8060(-3573, 22319) | 8831(-3841, 24471) | 9.57 | 73.35(-32.64, 203.45) | 53.1(-23.08, 146.99) | -0.74(-0.86, -0.62) |
| Azerbaijan | 2188(-851, 9060) | 2744(-1157, 10826) | 25.40 | 39.04(-15.26, 161.87) | 23.45(-9.87, 92.21) | -1.33(-1.61, -1.06) |
| Bahamas | 20(-7, 93) | 41(-14, 186) | 107.74 | 12.13(-4.31, 57) | 9.5(-3.13, 42.84) | -0.45(-0.58, -0.32) |
| Bahrain | 46(-19, 196) | 123(-47, 555) | 166.92 | 25.01(-10.39, 109) | 12.67(-4.89, 57.85) | -2.72(-3.01, -2.44) |
| Bangladesh | 3664(-1477, 14362) | 7122(-3258, 26311) | 94.36 | 7.3(-2.96, 28.49) | 4.93(-2.26, 18.08) | -1.25(-1.38, -1.13) |
| Barbados | 19(-7, 91) | 30(-12, 141) | 62.07 | 6.88(-2.51, 33.49) | 5.99(-2.39, 27.59) | -0.22(-0.33, -0.11) |
| Belarus | 2100(-880, 6892) | 1598(-691, 5565) | -23.89 | 15.87(-6.62, 52.18) | 9.98(-4.32, 34.66) | -2.28(-2.55, -2.01) |
| Belgium | 10768(-4170, 33232) | 8923(-3409, 28046) | -17.13 | 73.39(-28.37, 226.89) | 41.96(-16.07, 131.15) | -1.59(-1.76, -1.43) |
| Belize | 7(-3, 32) | 26(-12, 120) | 270.56 | 7.29(-3.45, 33.49) | 7.97(-3.9, 37.47) | 0.33(-0.16, 0.82) |
| Benin | 135(-46, 588) | 344(-112, 1414) | 153.55 | 6.65(-2.28, 28.92) | 6.33(-2.09, 25.74) | 0.05(-0.10, 0.20) |
| Bermuda | 20(-7, 86) | 22(-8, 90) | 9.58 | 31.44(-11.51, 134.91) | 16.87(-6.32, 69.2) | -1.81(-1.99, -1.64) |
| Bhutan | 25(-13, 102) | 59(-31, 219) | 131.51 | 9.09(-4.43, 36.73) | 9.35(-4.95, 34.73) | 0.19(0.07, 0.31) |
| Bolivia (Plurinational State of) | 629(-196, 2532) | 1417(-467, 6051) | 125.45 | 18.64(-5.83, 75.11) | 15.1(-4.97, 64.89) | -0.68(-0.79, -0.57) |
| Bosnia and Herzegovina | 2250(-1034, 9301) | 2800(-1350, 11763) | 24.42 | 49.01(-22.5, 203.34) | 45.85(-22.1, 192.27) | -0.12(-0.25, 0.01) |
| Botswana | 109(-39, 456) | 256(-95, 1121) | 135.15 | 17.77(-6.38, 74.9) | 15.87(-5.97, 68.61) | -0.68(-1.05, -0.32) |
| Brazil | 17523(-8075, 48083) | 38485(-17910, 108412) | 119.62 | 18.39(-8.5, 50.41) | 15.07(-7.01, 42.45) | -0.68(-0.76, -0.59) |
| Brunei Darussalam | 10(-3, 45) | 22(-7, 97) | 120.24 | 9.38(-3.05, 43.72) | 5.99(-1.91, 26.66) | -1.06(-1.26, -0.87) |
| Bulgaria | 4389(-2051, 13920) | 4853(-2188, 14781) | 10.58 | 35.33(-16.52, 111.86) | 38.27(-17.26, 116.51) | 0.58(0.44, 0.72) |
| Burkina Faso | 239(-92, 946) | 552(-272, 2223) | 130.92 | 5.24(-2.03, 20.74) | 5.66(-2.8, 22.51) | 0.51(0.33, 0.69) |
| Burundi | 210(-86, 963) | 334(-134, 1590) | 58.63 | 8.52(-3.48, 39.1) | 6.1(-2.47, 29.36) | -1.44(-1.69, -1.19) |
| Cabo Verde | 33(-14, 144) | 96(-36, 401) | 194.03 | 14.38(-6, 63.05) | 21.5(-7.96, 90.66) | 0.99(0.70, 1.27) |
| Cambodia | 611(-179, 2624) | 1658(-554, 7682) | 171.56 | 12.55(-3.72, 53.2) | 12.52(-4.19, 57.83) | -0.04(-0.12, 0.03) |
| Cameroon | 420(-130, 1685) | 1339(-427, 5703) | 218.55 | 8.87(-2.8, 35.53) | 9.84(-3.17, 41.29) | 0.44(0.35, 0.52) |
| Canada | 10776(-4362, 37866) | 12404(-5166, 42133) | 15.11 | 33.67(-13.63, 118.44) | 17.31(-7.24, 58.97) | -2.05(-2.21, -1.89) |
| Central African Republic | 161(-49, 855) | 278(-83, 1297) | 71.97 | 12.27(-3.68, 64.45) | 10.36(-3.08, 47.2) | -0.58(-0.64, -0.52) |
| Chad | 141(-48, 616) | 446(-157, 2026) | 216.23 | 4.85(-1.64, 21.06) | 7.3(-2.56, 33.12) | 1.58(1.46, 1.70) |
| Chile | 1364(-482, 5692) | 2396(-859, 9907) | 75.57 | 13.14(-4.65, 54.85) | 9.35(-3.35, 38.73) | -0.86(-0.96, -0.76) |
| China | 287251(-142922, 774059) | 695845(-358143, 1914504) | 142.24 | 31.91(-15.79, 85.86) | 32.3(-16.56, 88.8) | 0.05(-0.10, 0.20) |
| Colombia | 3946(-1450, 14960) | 7216(-2527, 26921) | 82.85 | 21.09(-7.78, 79.99) | 13.09(-4.59, 48.82) | -1.77(-1.87, -1.66) |
| Comoros | 14(-6, 64) | 34(-13, 159) | 138.30 | 6.68(-2.68, 29.76) | 6.58(-2.45, 30.54) | -0.25(-0.40, -0.10) |
| Congo | 166(-72, 759) | 369(-142, 1704) | 122.62 | 14.15(-6.06, 64.3) | 12.01(-4.58, 54.79) | -0.72(-0.90, -0.53) |
| Cook Islands | 4(-1, 16) | 6(-2, 25) | 58.90 | 28.61(-9.81, 121.94) | 22.59(-7.54, 95.96) | -0.78(-0.85, -0.70) |
| Costa Rica | 235(-85, 911) | 480(-160, 1874) | 103.88 | 13.33(-4.83, 51.56) | 8.7(-2.91, 33.98) | -1.45(-1.61, -1.29) |
| Croatia | 4069(-1632, 12312) | 3814(-1611, 11434) | -6.27 | 62.88(-25.18, 190.14) | 46.05(-19.39, 137.7) | -0.84(-0.95, -0.73) |
| Cuba | 1122(-315, 4842) | 1948(-590, 8619) | 73.60 | 10.9(-3.06, 47.04) | 10.11(-3.06, 44.72) | -0.03(-0.13, 0.08) |
| Cyprus | 29(-13, 96) | 62(-27, 209) | 114.06 | 3.58(-1.68, 11.99) | 2.98(-1.31, 10.12) | -0.07(-0.28, 0.14) |
| Czechia | 17355(-7157, 50967) | 13575(-5588, 41582) | -21.78 | 129.61(-53.42, 380.73) | 65.13(-26.8, 197.87) | -2.24(-2.31, -2.17) |
| Côte d'Ivoire | 200(-70, 940) | 533(-173, 2422) | 166.75 | 4.58(-1.62, 21.68) | 4.36(-1.42, 19.94) | -0.42(-0.58, -0.26) |
| Democratic People's Republic of Korea | 5810(-1846, 25959) | 10781(-4114, 44074) | 85.57 | 32.72(-10.5, 146.07) | 31.5(-12.02, 128.6) | 0.06(-0.06, 0.17) |
| Democratic Republic of the Congo | 1430(-661, 6994) | 3163(-1365, 16089) | 121.12 | 8.32(-3.82, 40.51) | 7.64(-3.26, 38.98) | -0.27(-0.55, 0.02) |
| Denmark | 5838(-2716, 16173) | 5127(-2231, 14101) | -12.18 | 78.57(-36.62, 218.69) | 44.6(-19.55, 123.37) | -1.70(-1.8, -1.61) |
| Djibouti | 10(-3, 44) | 61(-20, 273) | 499.85 | 6.49(-2.21, 28.14) | 8.36(-2.74, 37.52) | 0.84(0.79, 0.89) |
| Dominica | 8(-3, 33) | 12(-4, 52) | 55.92 | 13.12(-4.95, 55.27) | 13.98(-4.86, 60.49) | 0.36(0.23, 0.48) |
| Dominican Republic | 327(-93, 1381) | 1066(-328, 4748) | 226.20 | 8.28(-2.37, 35.02) | 10.41(-3.22, 46.4) | 1.14(1.01, 1.27) |
| Ecuador | 618(-254, 2591) | 1480(-602, 6118) | 139.36 | 11.01(-4.52, 46.17) | 8.95(-3.63, 36.96) | -0.70(-0.99, -0.41) |
| Egypt | 583(-236, 1925) | 2821(-1107, 9990) | 383.54 | 1.78(-0.72, 5.9) | 3.98(-1.56, 14) | 3.57(3.08, 4.06) |
| El Salvador | 326(-141, 1416) | 673(-279, 3009) | 106.55 | 10.54(-4.57, 45.64) | 11.04(-4.57, 49.51) | -0.04(-0.19, 0.12) |
| Equatorial Guinea | 23(-12, 97) | 73(-30, 334) | 212.79 | 10.78(-5.36, 44.68) | 12.75(-5.19, 58.17) | 0.76(0.59, 0.94) |
| Eritrea | 108(-46, 476) | 270(-109, 1189) | 149.66 | 7.68(-3.25, 33.46) | 8.2(-3.27, 36.64) | 0.05(-0.11, 0.21) |
| Estonia | 1694(-713, 5197) | 1047(-434, 3331) | -38.17 | 82.64(-34.72, 254.22) | 42.12(-17.54, 134.08) | -2.3(-2.42, -2.17) |
| Eswatini | 58(-18, 268) | 147(-40, 687) | 155.00 | 18.19(-5.69, 84.75) | 22.91(-6.18, 105.77) | 1.10(0.52, 1.68) |
| Ethiopia | 2980(-1252, 8894) | 3442(-1418, 10318) | 15.47 | 13.36(-5.72, 39.62) | 7.39(-3.06, 22.64) | -2.25(-2.51, -2.00) |
| Fiji | 34(-13, 153) | 61(-24, 286) | 80.37 | 8.64(-3.41, 39) | 7.5(-2.99, 35.03) | -0.61(-0.79, -0.44) |
| Finland | 3907(-1618, 11768) | 4130(-1683, 11981) | 5.70 | 56.14(-23.25, 169.11) | 34.86(-14.23, 101.04) | -1.41(-1.51, -1.31) |
| France | 33885(-14273, 107925) | 45217(-18035, 141756) | 33.44 | 45.18(-19.06, 143.78) | 38.29(-15.28, 119.86) | -0.25(-0.45, -0.04) |
| Gabon | 80(-25, 368) | 157(-44, 733) | 95.29 | 13.43(-4.18, 61.54) | 13.58(-3.93, 62.7) | 0(-0.05, 0.06) |
| Gambia | 13(-5, 55) | 36(-16, 155) | 186.76 | 3.36(-1.37, 14.66) | 3.5(-1.57, 15) | -0.05(-0.21, 0.10) |
| Georgia | 3275(-1407, 15156) | 2505(-1082, 11020) | -23.52 | 50.43(-21.66, 233.47) | 44.03(-19.09, 193.6) | 0.74(0.34, 1.14) |
| Germany | 55498(-24000, 166872) | 59320(-26236, 175150) | 6.89 | 46.65(-20.16, 140.18) | 34.39(-15.2, 102.17) | -0.84(-0.92, -0.77) |
| Ghana | 421(-160, 1561) | 1219(-435, 4730) | 189.69 | 6.44(-2.46, 23.68) | 7.12(-2.54, 27.56) | 0.56(0.46, 0.66) |
| Greece | 11786(-5739, 32377) | 13936(-6647, 38439) | 18.24 | 78.58(-38.27, 215.78) | 67.39(-32.24, 186) | -0.49(-0.54, -0.45) |
| Greenland | 78(-31, 283) | 89(-37, 332) | 14.27 | 206.78(-82.01, 753.1) | 117.25(-49.2, 439.71) | -1.87(-2.01, -1.73) |
| Grenada | 6(-2, 29) | 9(-3, 39) | 46.38 | 9.53(-3.09, 43.35) | 7.69(-2.51, 32.69) | -0.31(-0.62, -0.01) |
| Guam | 21(-9, 104) | 42(-16, 185) | 95.88 | 26.11(-11.11, 127.21) | 19.98(-7.79, 88.59) | -0.35(-0.62, -0.08) |
| Guatemala | 456(-152, 1831) | 881(-288, 3722) | 93.14 | 12.12(-4.07, 48.68) | 7.67(-2.51, 32.39) | -2.04(-2.3, -1.78) |
| Guinea | 240(-102, 1010) | 534(-209, 2291) | 122.69 | 7(-2.97, 29.39) | 8.91(-3.56, 38.37) | 0.97(0.84, 1.10) |
| Guinea-Bissau | 42(-13, 181) | 76(-28, 303) | 82.93 | 9.72(-3.09, 42.64) | 9.38(-3.33, 37.64) | 0.21(0.05, 0.38) |
| Guyana | 21(-8, 91) | 35(-14, 160) | 68.16 | 5.08(-2.05, 22.63) | 4.93(-1.94, 22.73) | 0.27(0.03, 0.51) |
| Haiti | 384(-128, 1782) | 658(-211, 3310) | 71.31 | 10.89(-3.64, 50.6) | 8.34(-2.68, 41.99) | -0.68(-0.78, -0.58) |
| Honduras | 344(-174, 1309) | 1509(-773, 6099) | 338.26 | 15.58(-7.86, 59.6) | 22.79(-11.71, 92.28) | 1.51(1.37, 1.65) |
| Hungary | 14845(-6591, 43037) | 15527(-6314, 44927) | 4.60 | 104.27(-46.32, 302.15) | 86.6(-35.2, 250.3) | -0.65(-0.85, -0.46) |
| Iceland | 25(-11, 84) | 38(-16, 127) | 50.42 | 9.29(-3.99, 30.71) | 6.96(-2.93, 23.18) | -0.74(-0.9, -0.58) |
| India | 32493(-16125, 87809) | 91143(-42856, 249416) | 180.50 | 6.15(-3.07, 16.67) | 7.15(-3.36, 19.54) | 0.42(0.28, 0.56) |
| Indonesia | 8827(-4075, 24972) | 27967(-11564, 82836) | 216.84 | 8.16(-3.75, 23.31) | 10.77(-4.41, 32.04) | 0.84(0.77, 0.91) |
| Iran (Islamic Republic of) | 3758(-1895, 10250) | 9983(-5024, 26630) | 165.64 | 13.04(-6.62, 36.04) | 12.34(-6.2, 33.11) | 0.14(0.01, 0.28) |
| Iraq | 1105(-488, 4651) | 3831(-1616, 16934) | 246.71 | 13.49(-5.94, 56.74) | 15.36(-6.49, 67.89) | 0.26(0.16, 0.37) |
| Ireland | 2993(-1279, 8508) | 3267(-1338, 9597) | 9.17 | 73.79(-31.54, 209.57) | 42.34(-17.35, 124.56) | -1.49(-1.62, -1.37) |
| Israel | 1254(-547, 3821) | 2226(-967, 6664) | 77.55 | 26.58(-11.6, 81.13) | 18.84(-8.14, 56.38) | -1.00(-1.17, -0.82) |
| Italy | 54474(-29086, 153266) | 48509(-26277, 130782) | -10.95 | 63.51(-33.92, 178.75) | 36.49(-19.67, 98.11) | -1.80(-1.85, -1.75) |
| Jamaica | 213(-78, 892) | 350(-133, 1533) | 63.79 | 12.54(-4.6, 52.35) | 11.34(-4.32, 49.78) | -0.53(-0.95, -0.10) |
| Japan | 15339(-6963, 38207) | 24004(-10992, 60118) | 56.49 | 8.93(-4.05, 22.24) | 6.78(-3.09, 16.91) | -0.91(-1.02, -0.81) |
| Jordan | 254(-105, 806) | 1038(-458, 3252) | 307.89 | 16.72(-6.91, 53.12) | 12.41(-5.43, 39.07) | -0.83(-1.05, -0.61) |
| Kazakhstan | 13866(-4660, 50361) | 6577(-2399, 24386) | -52.57 | 100.78(-33.86, 366.63) | 33.56(-12.23, 124.47) | -3.60(-3.76, -3.44) |
| Kenya | 189(-89, 531) | 892(-414, 2450) | 370.70 | 2.15(-1.01, 6.02) | 3.51(-1.62, 9.64) | 1.75(1.53, 1.96) |
| Kiribati | 4(-2, 17) | 9(-4, 44) | 130.74 | 9.95(-4.64, 43.09) | 11.46(-4.35, 53.95) | 0.45(0.36, 0.55) |
| Kuwait | 53(-23, 167) | 129(-55, 425) | 142.92 | 8.01(-3.55, 25.28) | 4.03(-1.75, 13.06) | -1.83(-2.18, -1.47) |
| Kyrgyzstan | 1803(-553, 7313) | 1139(-375, 4495) | -36.83 | 56.93(-17.46, 231.42) | 21.29(-7.03, 83.23) | -2.86(-3.15, -2.58) |
| Lao People's Democratic Republic | 315(-134, 1342) | 575(-216, 2326) | 82.69 | 13.98(-5.98, 59.35) | 11.58(-4.36, 46.66) | -0.66(-0.72, -0.60) |
| Latvia | 3035(-1406, 9418) | 1762(-833, 5456) | -41.96 | 84.58(-39.13, 262.78) | 49.24(-23.26, 152.47) | -1.87(-2.03, -1.72) |
| Lebanon | 589(-181, 2488) | 1321(-525, 6022) | 124.34 | 25.75(-7.82, 109.67) | 22.49(-8.97, 102.65) | 0.39(0.05, 0.74) |
| Lesotho | 133(-78, 578) | 386(-218, 1626) | 189.34 | 15.08(-8.88, 65.71) | 32.37(-18.27, 136.34) | 3.09(2.68, 3.5) |
| Liberia | 80(-32, 373) | 146(-50, 644) | 83.30 | 6.69(-2.72, 31.27) | 6.4(-2.18, 28.13) | 0.21(-0.08, 0.49) |
| Libya | 451(-184, 2155) | 1350(-580, 6304) | 199.34 | 22.94(-9.45, 109.73) | 23.68(-10.16, 109.75) | 0.31(0.11, 0.51) |
| Lithuania | 2021(-731, 6737) | 1469(-579, 5005) | -27.34 | 44.53(-16.06, 148.41) | 28.19(-11.14, 96.21) | -1.6(-1.73, -1.46) |
| Luxembourg | 514(-221, 1413) | 564(-261, 1537) | 9.87 | 96.92(-41.73, 267.53) | 55.03(-25.47, 149.82) | -1.65(-1.79, -1.51) |
| Madagascar | 354(-108, 1460) | 750(-254, 3298) | 111.67 | 6.45(-1.96, 26.73) | 5.73(-1.93, 25.04) | -0.33(-0.46, -0.20) |
| Malawi | 92(-42, 387) | 217(-89, 911) | 135.43 | 2.21(-1, 9.29) | 2.62(-1.06, 11.14) | 0.35(0.10, 0.61) |
| Malaysia | 584(-239, 1927) | 1900(-769, 6351) | 225.52 | 6.07(-2.52, 19.96) | 6.41(-2.61, 21.49) | 0.03(-0.15, 0.21) |
| Maldives | 7(-3, 29) | 12(-4, 53) | 78.62 | 7.25(-2.82, 30.03) | 3.51(-1.04, 15.01) | -2.86(-3.01, -2.71) |
| Mali | 204(-71, 974) | 473(-182, 2165) | 131.45 | 4.79(-1.68, 22.82) | 4.99(-1.9, 22.81) | 0.41(0.28, 0.55) |
| Malta | 107(-43, 427) | 147(-59, 587) | 37.71 | 24.76(-9.92, 99.16) | 16.75(-6.76, 66.56) | -1.2(-1.31, -1.08) |
| Marshall Islands | 3(-1, 14) | 8(-2, 33) | 154.20 | 16.8(-5.55, 77.76) | 19.45(-5.54, 85.79) | 0.69(0.57, 0.81) |
| Mauritania | 76(-35, 295) | 157(-80, 661) | 106.51 | 7.31(-3.38, 28.39) | 7.06(-3.56, 29.87) | -0.11(-0.37, 0.15) |
| Mauritius | 69(-26, 308) | 119(-43, 540) | 71.94 | 8.96(-3.37, 39.93) | 6.3(-2.3, 28.75) | -1.44(-1.65, -1.22) |
| Mexico | 8250(-4085, 20927) | 12182(-6148, 30759) | 47.66 | 18.74(-9.27, 47.61) | 9.47(-4.77, 23.93) | -2.56(-2.69, -2.44) |
| Micronesia (Federated States of) | 10(-4, 45) | 18(-7, 88) | 76.53 | 20.21(-7.9, 87.93) | 22.42(-8.24, 106.17) | 0.43(0.38, 0.48) |
| Monaco | 35(-17, 130) | 60(-28, 235) | 69.95 | 59.34(-27.73, 218.89) | 71(-33.33, 279.01) | 0.75(0.41, 1.09) |
| Mongolia | 890(-364, 3512) | 1430(-553, 5593) | 60.64 | 80.89(-33.23, 320.07) | 56.15(-21.72, 219.8) | -1.64(-1.83, -1.44) |
| Montenegro | 302(-144, 1157) | 470(-225, 1745) | 55.57 | 46.15(-22.02, 177.29) | 47.68(-22.82, 176.93) | 0.28(0.09, 0.47) |
| Morocco | 2794(-1246, 10410) | 7148(-3354, 25507) | 155.79 | 19.02(-8.48, 70.52) | 19.77(-9.24, 70.6) | 0.18(0.01, 0.34) |
| Mozambique | 273(-81, 1150) | 689(-207, 2864) | 152.11 | 4.49(-1.34, 18.83) | 5.93(-1.81, 24.91) | 1.46(1.27, 1.64) |
| Myanmar | 3336(-1249, 15253) | 5229(-2102, 22930) | 56.73 | 13.34(-5.05, 60.26) | 10.19(-4.13, 44.67) | -1(-1.06, -0.94) |
| Namibia | 40(-16, 189) | 96(-34, 418) | 140.81 | 5.59(-2.27, 26.58) | 6.28(-2.27, 27.34) | 0.25(-0.05, 0.55) |
| Nauru | 2(0, 8) | 2(-1, 9) | 22.19 | 30.23(-9.86, 150.16) | 29.1(-8.14, 134.82) | -0.09(-0.12, -0.07) |
| Nepal | 857(-336, 3485) | 1928(-723, 7439) | 125.03 | 8.21(-3.23, 33.21) | 7.83(-2.95, 30.13) | -0.07(-0.34, 0.2) |
| Netherlands | 4723(-2061, 15455) | 5242(-2297, 16820) | 11.01 | 24.43(-10.67, 79.89) | 15.54(-6.83, 49.86) | -1.34(-1.46, -1.22) |
| New Zealand | 992(-347, 3807) | 1271(-468, 4685) | 28.08 | 25.59(-9.12, 98.27) | 15.51(-5.72, 56.4) | -1.63(-1.72, -1.54) |
| Nicaragua | 107(-38, 432) | 286(-95, 1157) | 167.56 | 6.51(-2.32, 26.31) | 5.64(-1.88, 22.93) | -0.36(-0.56, -0.17) |
| Niger | 135(-48, 588) | 369(-121, 1555) | 173.71 | 4.52(-1.63, 19.87) | 4.28(-1.42, 18.03) | 0.18(-0.02, 0.38) |
| Nigeria | 683(-352, 1873) | 1524(-763, 4167) | 123.12 | 1.47(-0.76, 4.03) | 1.57(-0.8, 4.28) | 0.41(0.32, 0.50) |
| Niue | 0(0, 2) | 0(0, 2) | 20.68 | 18.6(-6.02, 83.27) | 21.94(-7.1, 94.84) | 0.52(0.44, 0.60) |
| North Macedonia | 1474(-794, 4459) | 2485(-1203, 7611) | 68.59 | 73.2(-39.29, 221.72) | 71.83(-34.89, 220.27) | 0(-0.3, 0.31) |
| Northern Mariana Islands | 7(-2, 30) | 14(-4, 65) | 113.35 | 34.16(-10.44, 155.45) | 26.14(-7.79, 118.91) | -0.91(-0.97, -0.84) |
| Norway | 2504(-1094, 6846) | 3360(-1497, 9148) | 34.19 | 40.65(-17.72, 111.22) | 34.51(-15.34, 93.99) | -0.53(-0.74, -0.32) |
| Oman | 35(-14, 146) | 72(-27, 285) | 107.45 | 4.77(-1.92, 19.87) | 3.28(-1.25, 12.6) | -0.97(-1.28, -0.67) |
| Pakistan | 8369(-3339, 26706) | 21471(-8242, 70369) | 156.56 | 14.24(-5.65, 45.62) | 16.03(-6.17, 52.63) | 0.11(-0.17, 0.39) |
| Palau | 4(-1, 17) | 8(-3, 36) | 115.54 | 35.11(-13.49, 161.72) | 32.98(-12.01, 154.97) | -0.08(-0.14, -0.02) |
| Palestine | 218(-90, 799) | 593(-252, 2125) | 171.78 | 24.11(-9.93, 88.35) | 21.41(-9, 75.93) | -0.44(-0.64, -0.25) |
| Panama | 240(-77, 1111) | 395(-126, 1910) | 64.98 | 15.68(-5.05, 72.82) | 8.96(-2.85, 43.31) | -1.83(-1.98, -1.67) |
| Papua New Guinea | 267(-89, 1239) | 805(-308, 3318) | 200.97 | 13.6(-4.6, 62.56) | 14.73(-5.56, 60.31) | 0.34(0.28, 0.40) |
| Paraguay | 174(-71, 614) | 692(-269, 2541) | 298.20 | 7.6(-3.14, 26.8) | 11.63(-4.51, 42.73) | 1.39(1.11, 1.67) |
| Peru | 1679(-527, 7969) | 3248(-1125, 15724) | 93.48 | 13.32(-4.15, 63.2) | 9.56(-3.31, 46.27) | -1.37(-1.75, -0.99) |
| Philippines | 3065(-1574, 8053) | 7921(-3890, 20356) | 158.39 | 9.49(-4.89, 24.94) | 8.93(-4.4, 23) | -0.48(-0.66, -0.31) |
| Poland | 21674(-9598, 60328) | 27051(-12388, 74013) | 24.81 | 49.22(-21.8, 136.85) | 38.3(-17.51, 104.81) | -0.9(-1.07, -0.72) |
| Portugal | 4613(-1863, 13445) | 6744(-2855, 20351) | 46.20 | 33.88(-13.66, 98.83) | 31.79(-13.37, 95.46) | -0.05(-0.23, 0.14) |
| Puerto Rico | 434(-155, 2023) | 416(-157, 1985) | -4.13 | 12(-4.29, 55.84) | 6.51(-2.44, 30.86) | -1.9(-2.07, -1.73) |
| Qatar | 21(-8, 91) | 93(-34, 434) | 337.91 | 16.82(-6.21, 73.58) | 7.95(-3.03, 35.79) | -2.41(-2.95, -1.87) |
| Republic of Korea | 12450(-5155, 39178) | 24765(-11996, 80687) | 98.91 | 38.09(-15.85, 119.44) | 26.15(-12.67, 85.08) | -1.55(-1.83, -1.27) |
| Republic of Moldova | 2437(-809, 9320) | 1776(-597, 6702) | -27.11 | 52.06(-17.29, 199.13) | 29.89(-10.04, 112.37) | -1.26(-1.57, -0.96) |
| Romania | 10109(-4723, 32839) | 12911(-5775, 42421) | 27.71 | 34.9(-16.31, 113.43) | 38.58(-17.04, 126.54) | 0.18(0.06, 0.30) |
| Russian | 137910(-67534, 356865) | 100245(-49151, 260309) | -27.31 | 73.41(-35.94, 190.06) | 41.91(-20.54, 108.95) | -2.04(-2.19, -1.89) |
| Rwanda | 314(-108, 1199) | 568(-173, 2392) | 80.84 | 10.06(-3.45, 38.32) | 8.2(-2.5, 34.29) | -1.46(-1.81, -1.11) |
| Saint Kitts and Nevis | 3(-1, 13) | 5(-2, 22) | 70.24 | 7.99(-3.44, 35.54) | 6.51(-2.71, 29.69) | -0.24(-0.44, -0.04) |
| Saint Lucia | 9(-3, 41) | 18(-6, 84) | 103.63 | 10.31(-3.55, 46.5) | 7.56(-2.63, 34.31) | -0.97(-1.12, -0.81) |
| Saint Vincent and the Grenadines | 5(-2, 20) | 9(-3, 38) | 80.18 | 6.9(-2.13, 28.47) | 6.14(-1.88, 25.93) | -0.1(-0.25, 0.05) |
| Samoa | 6(-2, 27) | 9(-4, 41) | 63.27 | 6.5(-2.73, 29.59) | 6.25(-2.95, 26.89) | -0.04(-0.14, 0.06) |
| San Marino | 14(-6, 52) | 14(-5, 52) | -4.04 | 41.79(-16.59, 152.45) | 20.55(-7.35, 78.72) | -1.38(-1.71, -1.04) |
| Sao Tome and Principe | 10(-5, 40) | 23(-10, 91) | 128.55 | 15.18(-6.91, 59.67) | 19.83(-8.91, 78.13) | 1.05(0.98, 1.12) |
| Saudi Arabia | 169(-76, 552) | 610(-314, 1816) | 261.34 | 2.65(-1.2, 8.73) | 2.59(-1.37, 7.59) | -0.08(-0.28, 0.12) |
| Senegal | 264(-104, 1222) | 687(-274, 3196) | 160.70 | 7.79(-3.08, 35.9) | 8.49(-3.41, 39.42) | 0.48(0.26, 0.71) |
| Serbia | 8069(-3753, 28544) | 9851(-4017, 34428) | 22.09 | 66.35(-30.81, 235.63) | 63.89(-26.04, 223.77) | -0.06(-0.23, 0.11) |
| Seychelles | 6(-3, 25) | 9(-4, 39) | 48.15 | 10.65(-4.58, 43.57) | 7.35(-2.95, 31.93) | -1.26(-1.42, -1.09) |
| Sierra Leone | 143(-58, 598) | 271(-105, 1119) | 89.65 | 6.81(-2.79, 28.31) | 6.79(-2.61, 27.95) | 0.46(0.22, 0.69) |
| Singapore | 171(-75, 744) | 270(-120, 1213) | 57.47 | 7.55(-3.32, 32.82) | 3.13(-1.39, 14.04) | -2.79(-2.92, -2.67) |
| Slovakia | 5940(-2584, 17401) | 5249(-2476, 15527) | -11.63 | 99.84(-43.44, 292.87) | 56(-26.44, 166.19) | -1.79(-1.86, -1.72) |
| Slovenia | 1860(-807, 5566) | 2077(-870, 6247) | 11.66 | 74.98(-32.57, 223.92) | 49.64(-20.84, 148.3) | -1.39(-1.54, -1.25) |
| Solomon Islands | 26(-9, 124) | 71(-20, 340) | 172.29 | 16.71(-5.35, 78.61) | 17.46(-4.99, 83.42) | 0.16(-0.02, 0.34) |
| Somalia | 144(-74, 651) | 333(-146, 1460) | 131.78 | 5.14(-2.7, 23.38) | 4.55(-2.06, 20.23) | -0.26(-0.32, -0.20) |
| South Africa | 5301(-2573, 15443) | 11863(-5354, 36501) | 123.80 | 23.59(-11.48, 68.84) | 23.97(-10.89, 73.6) | -0.10(-0.39, 0.20) |
| South Sudan | 167(-72, 746) | 274(-103, 1362) | 63.69 | 6.19(-2.65, 27.54) | 6.28(-2.39, 31.44) | 0.07(-0.06, 0.20) |
| Spain | 29185(-13158, 84090) | 36538(-15977, 102155) | 25.19 | 55.92(-25.19, 161.23) | 42.28(-18.42, 118.12) | -0.84(-0.98, -0.70) |
| Sri Lanka | 560(-223, 2410) | 1141(-399, 4810) | 103.63 | 4.83(-1.92, 20.81) | 4.07(-1.42, 17.22) | -0.30(-0.57, -0.03) |
| Sudan | 561(-230, 2391) | 1357(-558, 6024) | 142.04 | 5.61(-2.29, 23.99) | 6.21(-2.57, 27.88) | 0.51(0.43, 0.59) |
| Suriname | 26(-7, 116) | 61(-19, 268) | 130.27 | 9.86(-2.75, 43.16) | 9.24(-2.83, 40.53) | 0.07(-0.15, 0.30) |
| Sweden | 4464(-1712, 15534) | 4810(-1869, 17385) | 7.75 | 33.1(-12.68, 115.23) | 23.1(-9.01, 83.1) | -0.87(-1.08, -0.65) |
| Switzerland | 4795(-2261, 14013) | 5299(-2492, 15213) | 10.50 | 49.99(-23.52, 145.98) | 31.7(-14.85, 90.65) | -1.27(-1.39, -1.16) |
| Syrian Arab Republic | 683(-304, 2260) | 1513(-549, 4932) | 121.70 | 11.59(-5.15, 38.15) | 10.55(-3.84, 34.52) | -0.46(-0.58, -0.35) |
| Taiwan (Province of China) | 1157(-544, 3603) | 2671(-1238, 8262) | 130.77 | 6.81(-3.21, 21.18) | 6.39(-2.95, 19.74) | -0.24(-0.48, 0) |
| Tajikistan | 847(-397, 3604) | 864(-397, 3424) | 1.93 | 28.48(-13.37, 121.23) | 12.66(-5.67, 50.27) | -2.47(-2.73, -2.21) |
| Thailand | 5431(-2277, 19122) | 11056(-5497, 33423) | 103.57 | 14.32(-5.92, 50.43) | 10.31(-5.12, 31.3) | -1.67(-1.88, -1.46) |
| Timor-Leste | 22(-11, 99) | 69(-30, 302) | 210.10 | 7.16(-3.42, 30.7) | 7.76(-3.3, 33.88) | 0.26(0.05, 0.46) |
| Togo | 100(-33, 408) | 363(-127, 1527) | 263.62 | 7.58(-2.52, 30.72) | 8.65(-3.05, 36.43) | 0.5(0.44, 0.55) |
| Tokelau | 0(0, 2) | 1(0, 2) | 14.35 | 34.18(-11.59, 141.64) | 36.35(-13.78, 156.12) | 0.26(0.23, 0.29) |
| Tonga | 12(-5, 54) | 17(-7, 71) | 43.42 | 20.68(-9.16, 94.01) | 20.94(-9.05, 87.1) | 0(-0.15, 0.16) |
| Trinidad and Tobago | 70(-18, 324) | 136(-36, 632) | 93.18 | 8.2(-2.15, 37.7) | 6.96(-1.84, 32.29) | -0.56(-0.67, -0.45) |
| Tunisia | 1283(-551, 4078) | 3149(-1276, 9861) | 145.38 | 24.23(-10.38, 77.07) | 22.57(-9.13, 70.7) | -0.49(-0.61, -0.36) |
| Turkey | 19397(-7511, 61329) | 31752(-13733, 93940) | 63.69 | 25.26(-9.61, 103.61) | 13.11(-4.96, 52.21) | -1.51(-1.75, -1.27) |
| Turkmenistan | 541(-206, 2223) | 604(-228, 2410) | 11.64 | 18.79(-7.53, 86.07) | 20.68(-8.78, 93.48) | -2.10(-2.53, -1.66) |
| Tuvalu | 1(-1, 6) | 2(-1, 10) | 66.22 | 51.13(-19.86, 161.35) | 32.54(-14.06, 96.26) | 0.31(0.28, 0.35) |
| Uganda | 371(-149, 1568) | 993(-371, 4406) | 167.87 | 5.42(-2.18, 22.82) | 6.1(-2.3, 26.96) | -0.25(-0.56, 0.05) |
| Ukraine | 47043(-18959, 166682) | 21481(-7879, 71668) | -54.34 | 65.26(-26.32, 231.58) | 29.13(-10.68, 98.3) | -2.99(-3.2, -2.78) |
| United Arab Emirates | 82(-32, 326) | 395(-172, 1577) | 384.91 | 14.52(-5.7, 56.3) | 8.76(-3.6, 34.52) | -0.68(-1.21, -0.15) |
| United Kingdom | 51983(-24028, 132399) | 41018(-19165, 104549) | -21.09 | 60.02(-27.72, 153.05) | 32.56(-15.16, 83.47) | -1.83(-1.91, -1.76) |
| United Republic of Tanzania | 749(-277, 3200) | 1861(-776, 8178) | 148.52 | 6.38(-2.36, 27.2) | 6.68(-2.79, 29.34) | 0.03(-0.02, 0.08) |
| United States of America | 172869(-80717, 448924) | 165270(-77586, 428816) | -4.40 | 57.24(-26.72, 148.55) | 28.54(-13.39, 73.96) | -2.38(-2.56, -2.20) |
| United States Virgin Islands | 11(-3, 49) | 14(-5, 66) | 31.50 | 11.97(-3.81, 53.96) | 8.58(-2.79, 39.44) | -0.84(-1.04, -0.65) |
| Uruguay | 1302(-352, 6168) | 1301(-353, 6234) | -0.06 | 34.65(-9.36, 164.03) | 25.92(-7.06, 124.04) | -0.98(-1.06, -0.89) |
| Uzbekistan | 3496(-1331, 14118) | 3321(-1369, 13936) | -5.01 | 28.37(-10.79, 114.36) | 11.05(-4.56, 46.39) | -2.97(-3.28, -2.65) |
| Vanuatu | 10(-5, 40) | 28(-14, 126) | 190.99 | 14.31(-7.14, 58.63) | 14.84(-7.36, 65.76) | 0.11(0.05, 0.17) |
| Venezuela (Bolivarian Republic of) | 2340(-874, 10012) | 6122(-2214, 26654) | 161.69 | 22.54(-8.42, 96.47) | 19.85(-7.18, 86.66) | -0.44(-0.56, -0.32) |
| Viet Nam | 4611(-1669, 18892) | 12961(-5231, 57292) | 181.0989574 | 11.01(-4, 44.83) | 12.02(-4.84, 53.32) | 0.22(0.17, 0.27) |
| Yemen | 500(-209, 2195) | 1429(-661, 6085) | 185.6171988 | 9.11(-3.76, 40.02) | 9.18(-4.26, 39.18) | 0.22(0.12, 0.32) |
| Zambia | 244(-110, 1102) | 847(-320, 3749) | 246.784079 | 7.87(-3.53, 34.96) | 10.57(-4.07, 47.04) | 0.79(0.68, 0.90) |
| Zimbabwe | 566(-211, 2476) | 1215(-487, 5534) | 114.7344632 | 13.1(-4.85, 57.31) | 15.49(-6.11, 71.25) | 0.73(0.35, 1.11) |

UI：uncertainty intervals；EAPC: estimated annual percentage change

Supplementary Table S3 The mortality number and rates of lung cancer attributed to residential radon exposure in 1990 and 2021 across 5 SDI regions, and the trends from 1990 to 2021

| SDI | All ages mortality number | | | Age-standard mortality rate per 100000 population | | |
| --- | --- | --- | --- | --- | --- | --- |
|  | 1990(95% UI) | 2021(95% UI) | percentage change,% | 1990(95% UI) | 2021(95% UI) | EAPC(95% CI) |
| High | 19339(-9219, 49976) | 24685(-11977, 63910) | 27.64 | 1.74(-0.83, 4.50) | 1.14(-0.55, 2.95) | -0.63(-1.24, -0.02) |
| High-middle | 19367(-9534, 49901) | 30142(-14673, 82303) | 55.64 | 1.92(-0.95, 4.95) | 1.50(-0.73, 4.11) | -0.04(-0.33, 0.25) |
| Middle | 7841(-3994, 20306) | 20785(-11180, 53911) | 165.07 | 0.78(-0.39, 2.01) | 0.79(-0.43, 2.05) | 1.06(0.61 to 1.5) |
| Low-middle | 2010(-860, 5056) | 5114(-2333, 13066) | 154.49 | 0.34(-0.14, 0.84) | 0.36(-0.17, 0.92) | 0.85(0.14 to 1.57) |
| Low | 608(-294, 1705) | 1340(-679, 3479) | 120.50 | 0.27(-0.13, 0.77) | 0.27(-0.14, 0.71) | -0.09(-1.24 to 1.07) |

UI：uncertainty intervals；EAPC: estimated annual percentage change

Supplementary Table S4 The DALYs number and rates of lung cancer attributed to residential radon exposure in 1990 and 2021 across 5 SDI regions, and the trends from 1990 to 2021

| SDI | All ages DALYs number | | | Age-standard DALYs rate per 100000 population | | |
| --- | --- | --- | --- | --- | --- | --- |
|  | 1990(95% UI) | 2021(95% UI) | percentage change,% | 1990(95% UI) | 2021(95% UI) | EAPC(95% CI) |
| High | 467874(-222922, 1209466) | 508399(-246354, 1315798) | 8.66 | 43.25(-20.62, 111.88) | 25.42(-12.28, 65.81) | -1.05(-1.59, -0.51) |
| High-middle | 532349(-260871, 1378406) | 704755(-344325, 1916123) | 32.39 | 51.17(-25.12, 132.50) | 35.31(-17.29, 96.07) | -0.54(-0.83, -0.24) |
| Middle | 221402(-113433, 575327) | 505335(-274051, 1316530) | 128.24 | 19.71(-10.07, 51.09) | 18.255(-9.88, 47.45) | 0.64(0.3, 0.98) |
| Low-middle | 57456(-24718, 144666) | 139386(-63150, 357200) | 142.60 | 8.46(-3.71, 21.71) | 9.12(-4.14, 23.32) | 0.74(0.15, 1.34) |
| Low | 17431(-8423, 48478) | 37979(-19364, 98852) | 117.88 | 7.03(-3.40, 19.72) | 6.82(-3.46, 6.82) | -0.19(-1.19, 0.82) |

UI：uncertainty intervals；EAPC: estimated annual percentage change


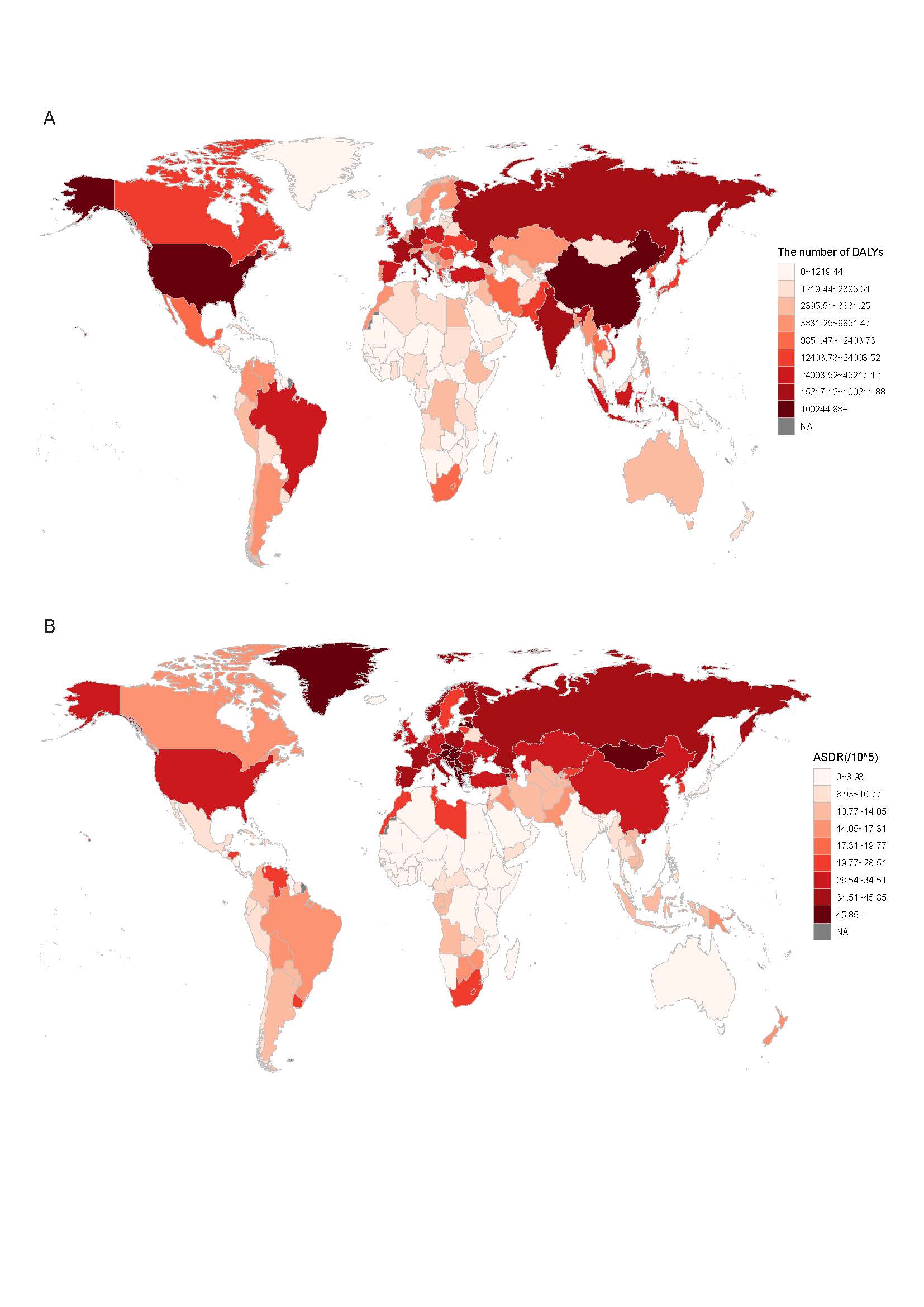


**Supplementary Fig.S1.** The DALYs (A) and ASDR (B) of LC attributed to residential radon exposure at global in 204 countries in 2021. DALYs, disability-adjusted life years; ASDR, age-standardized DALYs rate.


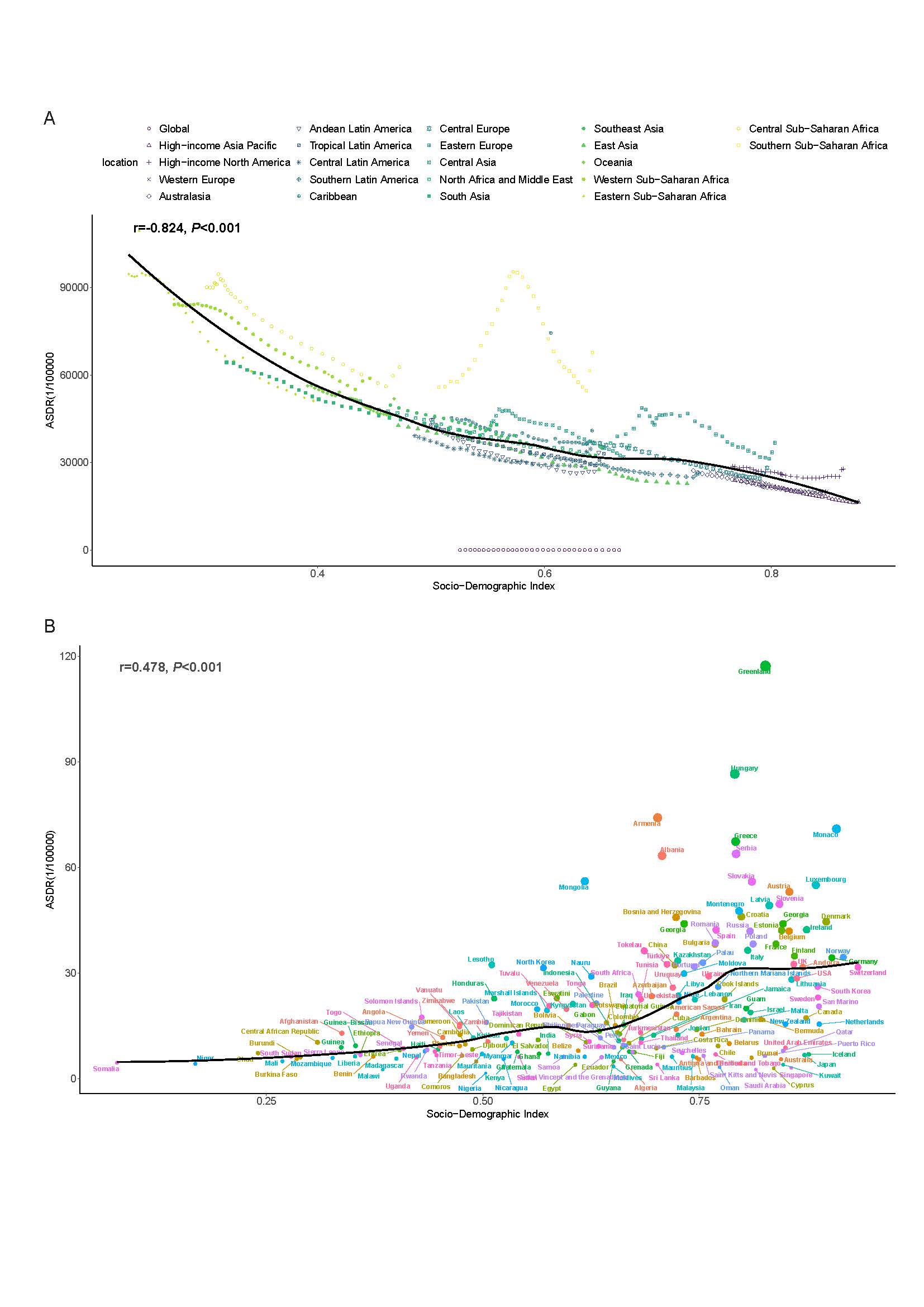


**Supplementary Fig.S2.** The associations between the SDI and ASDR of LC attributed to residential radon exposure across. (A) The associations between the SDI and ASDR in 21 regions and global according from 1990 to 2021; (B) The associations between the SDI and ASDR in 204 countries in 2021. The black line was the expected values based on the SDI and disease rates. SDI, socio-demographic index; ASDR, age-standardized DALYs rate; DALYs, disability-adjusted life years.
